# Assessment of bioclimatic change in Kazakhstan, end 20th—middle 21st centuries, according to the PRECIS prediction

**DOI:** 10.1371/journal.pone.0239514

**Published:** 2020-10-02

**Authors:** Maria Luisa Lopez Fernandez, Dauren Zhumabayev, Ricardo Marco Garcia, Kanat Baigarin, Maria Soledad Lopez Fernandez, Saken Baisholanov

**Affiliations:** 1 Department of Environmental Biology, Faculty of Sciences, University of Navarra, Pamplona, Navarra, Spain; 2 Laboratory of Green Energy and Environment, Nazarbayev University, Astana, Kazakhstan; 3 NURIS, Nazarbayev University, Astana, Kazakhstan; 4 ”National Conservation Initiative” Corporate Fund, Astana, Kazakhstan; 5 Instituto de Estudios Manchegos, Ciudad Real, Spain; 6 International Science Complex “Astana”, Astana, Kazakhstan; Universidade de Vigo, SPAIN

## Abstract

We evaluate bioclimatic changes in Kazakhstan from the end of the 20th century until the middle of the 21st century to offer natural resource managers a tool that facilitates their decision-making on measures to adapt agriculture and environmental care to foreseeable climate change. We use climatic data from the “Providing REgional Climates for Impact Studies” (PRECIS) prediction and study them following the Worldwide Bioclimatic Classification System (WBCS) of Rivas-Martínez. For three 25-year intervals (1980–2004, 2010–2034 and 2035–2059), we identify the continentality, macrobioclimates, bioclimates, bioclimatic variants, thermotypes, ombrotypes and isobioclimates of the study area. The results of the work allow us to: locate the territories where bioclimatic conditions will change, quantify the magnitude of the predicted climate changes, and determine the trends of predictable climate change. We present the results in maps, tables and graphs. For the 80-year interval, we identify 3 macroclimates, 3 bioclimatic variants, 10 bioclimates, 11 thermotypes, 10 ombrotypes and 43 isobioclimates. Some of those found bioclimates, thermotypes, ombrotypes and isobioclimates are only located in the E, SE and S mountains, where they occupy very small areas, that decrease in a generalized way as the 20th century progresses. Comparing the three successive periods, the following trends are observed: 36.2% of the territory increases in thermicity; 7.3% of the territory increases in continentality; 9.7% of the territory increases in annual aridity; 9.5% of the territory increases in summer aridity or mediterraneity; and generalized losses occur in the areas of all mountain isobioclimates. The climate change foreseen by the PRECIS model for the middle of the 21st century leads to bioclimatic homogenization, with 20.8% losses in bioclimatic diversity. We indicate on maps the locations of all the predicted bioclimatic changes; these maps may provide decision makers with a scientific basis to take necessary adaptation measures.

## Introduction

Predicted climate change threatens the socioeconomic balance of Kazakhstan. In fact, the agricultural sector uses 77% of the territory of Kazakhstan and occupies 25% of all labor resources in the country. In 2011, agricultural land totaled approximately 2 million square kilometers. In 2012, twelve million tons of wheat, three million tons of potatoes, eight million tons of vegetables and melons, and almost one million tons of meat were produced [1]. Agriculture in Kazakhstan is mainly rainfed, and therefore, it is very sensitive to precipitation rates [2]. The connections between climatic parameters and agricultural productivity in Kazakhstan between 1988 and 2008 were investigated [3] and a strong correlation between rainfall and crop productivity and an inverse pattern with temperature was found.

In the “National Human Development Report 2008”, [4], Kelimbetov (p. 2) affirms: “climate change is one of the most important problems of the century. The territory of Kazakhstan is located in climatic zones which are very sensitive to physical fluctuations in conditions. Climate change may disturb the natural balance and lead to irreversible environmental processes.” In the same report [4] (p. 3), Haoliang Xu, United Nations (UN) Resident Coordinator/United Nations Development Programme (UNDP) Resident Representative, asserts: “Sustainable human development in the modern world is impossible without the study and alleviation of climate change impact on the economy, communities and the environment”; and he continues:”Climate change has an acute impact on the poorest …, primarily the rural disadvantaged population, …, as climate change is more closely connected to agricultural problems… International experience shows that the climate change risks can be mitigated. …. This is especially important for a country such as Kazakhstan, where 9% of Gross Domestic Product (GDP) is derived from agriculture and is thus more exposed to climate change effects than many other countries.” To mitigate the effects of upcoming climate change and propose practical measures to resolve climate change problems, the first necessary step is to determine the magnitude, direction and location of climate change.

The International Journal of Biometeorology, published by Springer for the INTERNATIONAL SOCIETY OF BIOCLIMATOLOGY AND BIOMETEOROLOGY publishes studies examining the relationships between the atmospheric environment and living organisms [5–8]. Living organisms extend from single-cell organisms to plants and animals, including humans. The atmospheric environment includes both climatological and meteorological conditions, in both natural and artificial surroundings, for both historical or climate-change related analyses. Since 1995 Rivas-Martínez has been working on one of the particular aspects of bioclimatology, which he defines as an “ecological science”, studying the relationship between the climate and the distribution of the living species on the Earth. The aim of this subject is to determine the relation between certain numerical values of temperature and precipitation with the geographic distribution areas of single plant species as well as of plant communities”. [9–11]. The “Rivas-Martínez Worldwide Bioclimatic Classification System (WBCS)” has been the result of that work: “an easily applicable bioclimatic typology that shows an adjusted relationship between the vegetational model components and the climate values; given the high predictive value of bioclimatic units, they can be used in other sciences, in the programs of study and conservation of biodiversity and of "habitats”, in the forecast for obtaining agricultural and forestry resources, in the determination of future climatic and vegetative scenarios on Earth.” [10: 1a. Introduction].

The WBCS has already been applied in North America [12, 13]; in Mexico [14–19]; Central America, Dominican Republic [20]; South America [21], Venezuela [22], Bolivia [23, 24], Chile [25]; Europe [26], Iberian Peninsula [27, 28], Peninsular Spain [29–33], Canary Islands [34, 35], continental and insular Portugal [36–38], Italy [39, 40]; Asia, Far East Russia [41], Kazakhstan [42–44], Iran [45], Palestine [46]; Africa [47]; Oceania, Australia [48].

The method of Rivas-Martinez and colleagues observes and analyzes outcomes of vegetation climate interactions, i.e., vegetation needs with respect to temperature, precipitation and their annual rhythms, and collects them in their bioclimatic classification of the Earth. [49].

Projections of climatic conditions produced from any global circulation model (GCM) for any period into the future, 25, 50, or more years, can be used to produce bioclimatic maps. In addition, comparing the maps of successive periods in time, we can determine, assess, and locate the possible effects of climate change, the trends of change, and the changes in size and location of the bioclimatic units, as well as foresee their influence on vegetation and plant species, both wild and agricultural. This knowledge will allow adapting and mitigating the effects of climate change on the human population and its activities on a scientific basis. There are already several studies on climate change carried out with the WBCS methodology. [14, 50–58].

The WBCS [12, 49] has already been applied in Kazakhstan. In 2002, a bioclimatic map of Kazakhstan, with all the known and available climatic data issued by weather stations in Kazakhstan from the end of the 19th century until 1968 [59], has been published [42]. Now, in this work, we apply the WBCS to the climatic data of the PRECIS prediction—a modelling system made freely available to groups of developing countries- corresponding to three periods of 25 years from the end of the 20^th^ century until the middle of the 21^st^ century. Calculation of bioclimatic indexes in this region is done using temperature and precipitation values. According to IPCC projections seasonal precipitation in region will remain approximately the same except moderate increase in winter [60]. Whereas temperature increase is directly driven by emission scenario and could raise from 1 to 7 degrees. These trends of gradual temperature increase and relatively stable precipitation are confirmed in local studies [61]. The highest temperature increase is exhibited in RCP8.5 scenario, so the authors consider it as the most extreme climate change scenario. Because the authors motivation was investigating the impact of the most extreme climate change scenario, only RCP 8.5 scenario was used in this study. Knowing the impact of the most extreme scenario will allow decision-makers to calculate how far the effort will have to go to mitigate and adapt to the effects of predicted climate change. Other less extreme scenarios of climate change will require proportionally less adaptation efforts.

We reflect the results of the classification in tables, graphs and maps of continentality, macrobioclimates, bioclimates, variants, thermotypes, ombrotypes and isobioclimates for each of the periods considered. We compare the obtained results to assess the magnitude, location and trends of climate change foreseen by PRECIS.

Changes in weather conditions can affect different sectors of Kazakhstan’s economy: the energy system, ecosystem, health, tourism, water supply and agriculture [62]. The link between policy makers and managers and available data is the use of data from regional climate models and sectoral impact models to analyze the consequences of climate change [63]. Our results provide some of the necessary tools for policy makers and managers to study, foresee and decide, on a scientific basis, the measures leading to Kazakhstan’s adaptation to the foreseen climate change.

## Materials and methods

We consider as our material the study area, the worldwide bioclimatic classification system as the system to bioclimatically classify the data, the PRECIS projection climatic data used, and the program used to draw the maps. Our method involves the preparation of the PRECIS projection basic climate data, the calculation of climatic parameters and bioclimatic indices, the bioclimatic classification-identification, and the comparison between periods.

### Study area

Our study area is Kazakhstan, one of the Central Asian countries, which, with its 2.7 million km^2^, is the ninth largest country in the world. Its size is equivalent to that of Western Europe, and its population is almost 18 million inhabitants. Its latitudinal and longitudinal limits are 40°34´–55°26´ North and 46°28 ´–87°13´ East. It is bounded to the N by Russia, to the E by China, to the S by Kyrgyzstan, Uzbekistan and Turkmenistan, and to the W by Russia and the Caspian Sea. We use a grid cell resolution of 0.22° by 0.22°, equivalent to 24.3 km x 24.3 km, with an area for each cell of 590.9 km^2^. The whole of Kazakhstan includes 4569 of these cells.

### Worldwide bioclimatic classification system: A brief description

Rivas-Martínez and his school base their WBCS on “hundreds of thousands of releves sampled along boreal—tropical latitudinal and elevational gradients” [17], p. 3, as well as on the estimation of some factors that condition plant life and its distribution and that the authors consider as principles or axioms of the WBCS: reciprocity, photoperiod/latitude, continentality/oceanity, seasonality of rainfall, mediterraneity, types of deserts, oroclimates and orogenies. (For more information see [49], pp. 3–4):

The following bioclimatic units are considered in the WBCS: macrobioclimates, bioclimates, variants, thermotypes, ombrotypes and isobioclimates. To define all these hierarchical bioclimatic units, the WBCS uses a certain number of climatic parameters and bioclimatic indexes, carefully chosen and related to some of the many physiological demands of the plants throughout their life cycle. See Table 1: WBCS climatic parameters and bioclimatic indexes, which is a list of the main climatic parameters and bioclimatic indexes used in the WBCS.

**Table 1:** WBCS climatic parameters and bioclimatic indexes.

**Climatic parameters**	
T	average annual temperature in degrees centigrade
Ti	average monthly temperature, where i: 1 = January,…, 12 = December
M	average temperature of the maximums of the coldest month of the year
m	average temperature of the minimums of the coldest month of the year
Tmax	average temperature of the hottest month of the year, in degrees centigrade
Tmin	average temperature of the coldest month of the year, in degrees centigrade
Tp	positive annual temperature: total in tenths of degrees centigrade of the average monthly temperatures higher than 0°. ∑Ti (1–12) > 0°C, where i: 1 = January,…, 12 = December
Tps	positive temperature of the summer quarter, in tenths of degrees centigrade, if Ti>0
P	annual precipitation in millimeters or liters per square meter
Pi	monthly precipitation, where i: 1 = January,…, 12 = December
Pcm1	precipitation of the warmest four months of the year
Pcm2	precipitation of the four months following the warmest of the year
Pcm3	precipitation of the four months before the warmest of the year
Pp	positive annual precipitation (of the months with a Ti higher than 0°C). ∑ Pi, Ti > 0°
Ps	precipitation of the summer quarter
Pw	precipitation of the winter quarter
Pps	positive precipitation of the summer quarter. ∑ Ps, Ti > 0°C
Ppw	positive precipitation of the winter quarter. ∑ Pw, Ti > 0°C
Pss	precipitation for the warmest six months of the year
Psw	precipitation for the coldest six months of the year
**Bioclimatic indexes**	
Ic	Continentality/Oceanity Index: Annual thermal amplitude Ic = Tmax-Tmin, in degrees centigrade
Itc = Cti	Compensated Thermicity Index: It = (T + m + M) x 10 ±C
	(To calculate C, visit https://www.globalbioclimatics.org)
Io	Annual Ombrothermic Index: Io = (Pp / Tp) x 10
Iosi	Ombrothermic Index of any summer month: Iosi = (Psi/Tsi)10
Ios2	Ombrothermic Index of the warmest two-month period of the summer quarter (Tr3)
Iosc3 (= Ios3)	Compensable Ombrothermic Index for the summer quarter (Tr3), used to assess summer aridity
Iosc4 (= Ios4)	Compensable Ombrothermic Index for the four-month period resulting from adding to the summer quarter (Tr3) the immediately previous month. Used to assess summer aridity
Iod2	Ombrothermic index of the driest two months in the driest quarter of the year

Adapted from: Rivas-Martínez S, Rivas Sáenz S, Penas A. Worldwide Bioclimatic Classification System. Global Geobotany 2011;1: pp 5–6.

The WBCS is a hierarchical bioclimatic classification, with three levels. The 1st hierarchical level is that of macrobioclimates. The 2nd hierarchical level is that of bioclimates / bioclimatic variants: each macrobioclimate includes several bioclimates / variants. Finally, the 3rd hierarchical level is that of bioclimatic belts, which are sets of one thermotype and one ombrotype: each bioclimate/bioclimatic variant includes several bioclimatic belts. See S1 Fig “Bioclimatic synopsis of the earth”, with all the hierarchical bioclimatic units, as well as all of the bioclimatic parameters and bioclimatic index thresholds that define each of them. For the whole world, as seen in S1 Fig, the WBCS distinguishes 5 macrobioclimates, 28 bioclimates, 8 bioclimatic variants, 31 thermotypes and 9 ombrotypes ([49, 64]).

#### Isobioclimates

An isobioclimate is a unique bioclimatic space, defined by one bioclimate together with one bioclimatic belt (thermotype + ombrotype). Each isobioclimate is an elemental bioclimatic space, as perceived and distinguished by living vegetation. Each isobioclimate can be considered a natural culture chamber, a natural phytotron, whose "limiting walls" are the lower and upper thresholds that define each one of its components: bioclimate/variant and bioclimatic belt (thermotype and ombrotype). To name each isobioclimate, a phrase is used that includes bioclimate, together with a bioclimatic belt -thermotype + ombrotype-. Thus, for example: “Medc Sme Ari” is the isobioclimate "mediterranean desert—continental, supramediterranean, arid", which operates, for example, in Dzhezkazgan, Kazakhstan [64]. “Some four hundred isobioclimates are recognizable on the Earth, although only some three hundred have an appreciable territorial presence.” ([49], p. 16).

### Projection for climatic data

This paper uses climatic data provided by the regional model PRECIS (Providing REgional Climates for Impact Studies), developed by the Met Office Hadley Center [65]. In the PRECIS link, “https://www.gov.uk/dfid-research-outputs/the-hadley-centre-regional-climate-modelling-system-precis-providing-regional-climates-for-impacts-studies”, it is said: “The intention is to make this modelling system, PRECIS (Providing Regional Climates for Impacts Studies), freely available to groups of developing countries so that climate change scenarios can be developed at national centers of expertise”.

The lateral boundary conditions for HadGEM2-ES [66] under RCP8.5 emission scenario were made available by the Hadley Centre. These scenario run were carried out at Nazarbayev University Research and Innovation Systems, Kazakhstan, for the period 1960–2099 for the area that stretches from 46-86E and 35–55N. The resolution of the simulation is 0.22° by 0.22°, which allows good spatial details to be obtained over the region. So, each grid-cell (or point) has an area of 590.9 km2, and there are 4569 grid-cells (or points) for the whole of Kazakhstan.

Several parameters were extracted from the model: monthly mean of daily minimum and maximum temperature, monthly precipitation rate and spatial orography data used by the model.

Seasonal temperature and precipitation validation against CRU TS 3.23 [67] observational data is presented in S2 and S3 Figs, which are visualizations of PRECIS data and CRU TS 3.23 observations using Python software (the various datasets on the CRU website are provided for all to use, provided the source is acknowledged. Python is a free software language). S2 and S3 Figs are own elaboration for this article: see these figures in the Supporting Information. In general, the model represents temperature well, whereas precipitation is in moderate agreement with the observations. The latter seems to be a problem in the majority of the climate models.

### Preparation of the projection of basic climate data

From all the data—monthly average of the minimum and maximum temperatures, monthly rainfall, and spatial orographic data—corresponding to 13536 georeferenced grid-cells of 0.22° by 0.22° (590.9 km^2^) derived from the regional model PRECIS, which includes all of Central Asia, we extracted the 4569 georeferenced grid cells corresponding to Kazakhstan, with indication, for each of the points, of its latitudinal, longitudinal and altimetric values. In the QGIS 2.18.22 program, run on the operative system system OS X Yosemite, version 10.10.5, the political limits of Kazakhstan, obtained in: https://www.geoboundaries.org/, were used as clipping tool. Specifically, the download was made in: https://www.geoboundaries.org/data/geoBoundaries-2_0_0/KAZ/ADM0/. (GeoBoundaries is an open product: all boundaries are open and redistributable) [68]. Thus, we obtained a table of 4569 georeferenced grid cells for average monthly minimum temperatures, another table for average monthly maximum temperatures, and a third table for monthly rainfall, for each of the three study periods: 1980–2004, 2010–2034 and 2035–2059. Each table was accompanied by the corresponding geographic data (latitude, longitude and height) for each grid cell: that is to say, all of the 4569 cells, corresponding to Kazakhstan, were georeferenced.

For each of the three time periods under study, we prepared a basic data table, with the average monthly minimum temperatures, the average monthly maximum temperatures, the average monthly temperatures calculated as the average between the maximums and the minimums, and the monthly rainfall, for each of the 4569 georeferenced grid cells. In total, we prepared three basic data tables, one for each of the three study periods (see S1–S3 Tables), to perform the calculations of both climatic parameters and bioclimatic indexes to be used in the worldwide bioclimatic classification system WBCS [49].

### Calculation of climatic parameters and bioclimatic indexes

With the aid of the Excel software (Microsoft Excel 15.28, with OS X Yosemite Version 10.10.5) and the three basic data S1–S3 Tables, the 28 climatic parameters and bioclimatic indexes given in Table 1 have been calculated following the WBCS, for each study period, as well as the possible compensation of the summer aridity, (Ios_2_ ≤ 2), according to Io, Ios2, Iosc3 and Iosc4 ([49], p. 8; [64], paragraph 9). The results of these calculations have enabled the bioclimatic classification of the 4569 georeferenced grid cells of Kazakhstan territory through the identification of the bioclimatic units in each of the considered grid cells.

### Bioclimatic classification-identification

For the bioclimatic classification-identification of the 4569 georeferenced grid cells corresponding to Kazakhstan, and for each of the three periods of time considered, we have proceeded as follows:

#### Continentality

Due to the bioclimatic importance of the continentality, in addition to calculating the continentality index, Ic = Tmax-Tmin, for all the Kazakhstan grid cells, according to the thresholds of Table 2 ([49]), we have assigned them the corresponding types, subtypes and, in some cases, continentality levels.

**Table 2:** Types, subtypes and levels of continentality.

Types	Subtypes	Levels	Ic Values
1. Hyperoceanic (0–11)	Ultrahyperoceanic	1.1a. Strong	0–2.0
		1.1b. Weak	2.0–4.0
	Euhyperoceanic	1.2a. Strong	4.0–6.0
		1.2b. Weak	6.0–8.0
	Subhyperoceanic	1.3a. Strong	8.0–10.0
		1.3b. Weak	10.0–11.0
2. Oceanic (11–21)	Semihyperoceanic	2.1a. Strong	11.0–12.0
		2.1b. Weak	12.0–14.0
	Euoceanic	2.2a. Strong	14.0–15.0
		2.2b. Weak	15.0–17.0
	Semicontinental	2.3a. Weak	17.0–19.0
		2.3b. Strong	19.0–21.0
3. Continental (21–66)	Subcontinental	3.1a. Weak	21.0–24.0
		3.1b. Strong	24.0–28.0
	Eucontinental	3.2a. Weak	28.0–37.0
		3.2b. Strong	37.0–46.0
	Hypercontinental	3.3a. Weak	46.0–56.0
		3.3b. Strong	56.0–66.0

From: Rivas-Martínez S, Rivas Sáenz S, Penas A. Worldwide Bioclimatic Classification System. Global Geobotany 2011;1: p 7.

#### Macrobioclimates

First, we calculated T, M, Itc and Tp values as if the grid cells were at 200 m height, using the coefficients indicated in note (1) in S1 Fig: "Synopsis of the Bioclimatic Classification of the Earth”. For the identification of the macrobioclimates, we applied the information in the first column (corresponding to macrobioclimates) of the aforementioned S1 Fig, using the T, M, Itc and Tp values reduced to 200 m, and in addition, those of Tps, m, Pcm_1_, Pcm_2_, Pcm_3_, Pss, Psw, and Ic, as well as the summer aridity presence/absence.

#### Bioclimates

Once each grid-cell macroclimate was known, its bioclimate was identified by using Ic and Io values and by applying the second column of S1 Fig.

#### Bioclimatic variants

To recognize the variants that operate in the territory, the thermal and ombric peculiarities that define them have been analyzed. The steppe (Stp) variant, which appears in the mediterranean, temperate, boreal and polar macrobioclimates, requires the presence of three conditions: Ps ≥ Pw; 6.0 ≥ Io > 0.2: and Psi < 3T. The submediterranean variant, frequent in the temperate macrobioclimate and rare in the boreal and polar macrobioclimates, appears when during at least one summer month, Iosi: P < 2.8T. Grid cells that did not have these peculiarities were considered normal variants.

#### Thermotypes

To identify the thermotype of each point, it is necessary to know the following values: macrobioclimate, Ic, Tp and Itc.

Each macrobioclimate has its own threshold values for its thermotypes. For this reason, S1 Fig is ordered by macrobioclimates. For the mediterranean and temperate macrobioclimates, the table shows a call "(2)", which refers to the information in the lower part of the table: “(2) If Ic is greater than or equal to 21 (continental), or Itc <120, the thermotype must be calculated according to the Tp”. However, the boreal macrobioclimate thermotypes are directly identified by Tp intervals.

On a practical level, we ordered the mediterranean macrobioclimate grid cells by Ic. The grid cells with Ic greater than or equal to 21 were ordered by Tp and were classified according to Tp thresholds. The grid cells with Ic less than 21 were ordered by Itc: a) grid cells with Itc greater than or equal to 120 were classified by Itc thresholds; and b) grid cells with Itc less than 120 were ordered by Tp and were classified by their own thresholds of Tp.

Regarding the temperate macrobioclimate grid-cells, we followed the same steps as for the mediterranean macrobioclimate grid cells but used the thresholds for the temperate macrobioclimate, according to S1 Fig.

The boreal macroclimate grid cells were ordered directly by Tp and classified by their thresholds, according to S1 Fig.

#### Ombrotypes

The values of Io are used to identify ombrotypes. Now, since the ombrotype threshold values are the same for all macrobioclimates, to identify each grid cell ombrotype, the table is ordered by Io, and the ombrotypes are classified according to the S1 Fig thresholds.

#### Isobioclimates

We considered isobioclimates as the assemblage of a bioclimate plus a bioclimatic belt. To determine how many and which isobioclimates operated or will operate in Kazakhstan during the considered three periods of time, information on the bioclimate plus bioclimatic belt in each of the 4569 grid cells of Kazakhstan was collected in adjacent columns of an Excel file for each of the three periods. It was thus possible to determine how many and which isobioclimates operated or will operate in each of the periods. Gathering the information from the three periods, each isobioclimate could be assigned a key number, which would be easy to use when preparing distribution maps.

### Program for drawing bioclimatic maps

The Quantum Geographic Information System (QGIS 2.18.22) (link: https://www.qgis.org/en/site/forusers/download.html), a free geographic information program, run on operating system OS X Yosemite, version 10.10.5, is used to map the results found in the bioclimatic classification of each one of the 4569 georeferenced grid cells, for each one of the seven bioclimatic indicators, in each one of the three time periods (information collected in S4 Table). Since the grid cells were geo-referenced, the results of their bioclimatic classification could be immediately expressed in distribution maps. In the legend of each map appear the colors assigned to each value of the bioclimatic indicator represented in it. Thus, in QGIS, we have drawn a map for each of the bioclimatic indicators studied -continentality, macrobioclimates, bioclimates, bioclimatic variants, thermotypes, ombrotypes and isobioclimates- in each of the three time periods considered, 1980–2004, 2010–2034 and 2035–2059.

### Comparison between periods

For each bioclimatic unit, the obtained results for each of the three successive periods were tabulated, in absolute value and in %, and the numerical results were represented by graphs. Additionally, for each of the studied bioclimatic units, three maps were drawn, corresponding to the three 25-year periods considered: 1980–2004, 2010–2034 and 2035–2059. In this way, the trends of climate change could be deduced.

To determine the locations of the changes detected numerically for each bioclimatic unit, maps of the georeferenced grid cells that changed their classification from one period to another were produced. Likewise, to quantify the country bioclimatic stability, the points that remained constant from one period to another, and during all of the three study periods, were also represented on maps.

## Results

In this work on predicted climate change in Kazakhstan according to the PRECIS model, we studied seven bioclimatic units [44]: continentality, macrobioclimates, bioclimates, bioclimatic variants, thermotypes, ombrotypes and isobioclimates. The results appear in S4 Table “Bioclimatic Identification”. Finally, we comment on the results obtained for each of the bioclimatic units, collecting the information in tables, representing it in graphs, and illustrating it in maps. Thus, we see the extents, locations, changes and tendencies of change, in each of the bioclimatic units during the three successive periods of time considered.

### Continentality

**Fig 1:**
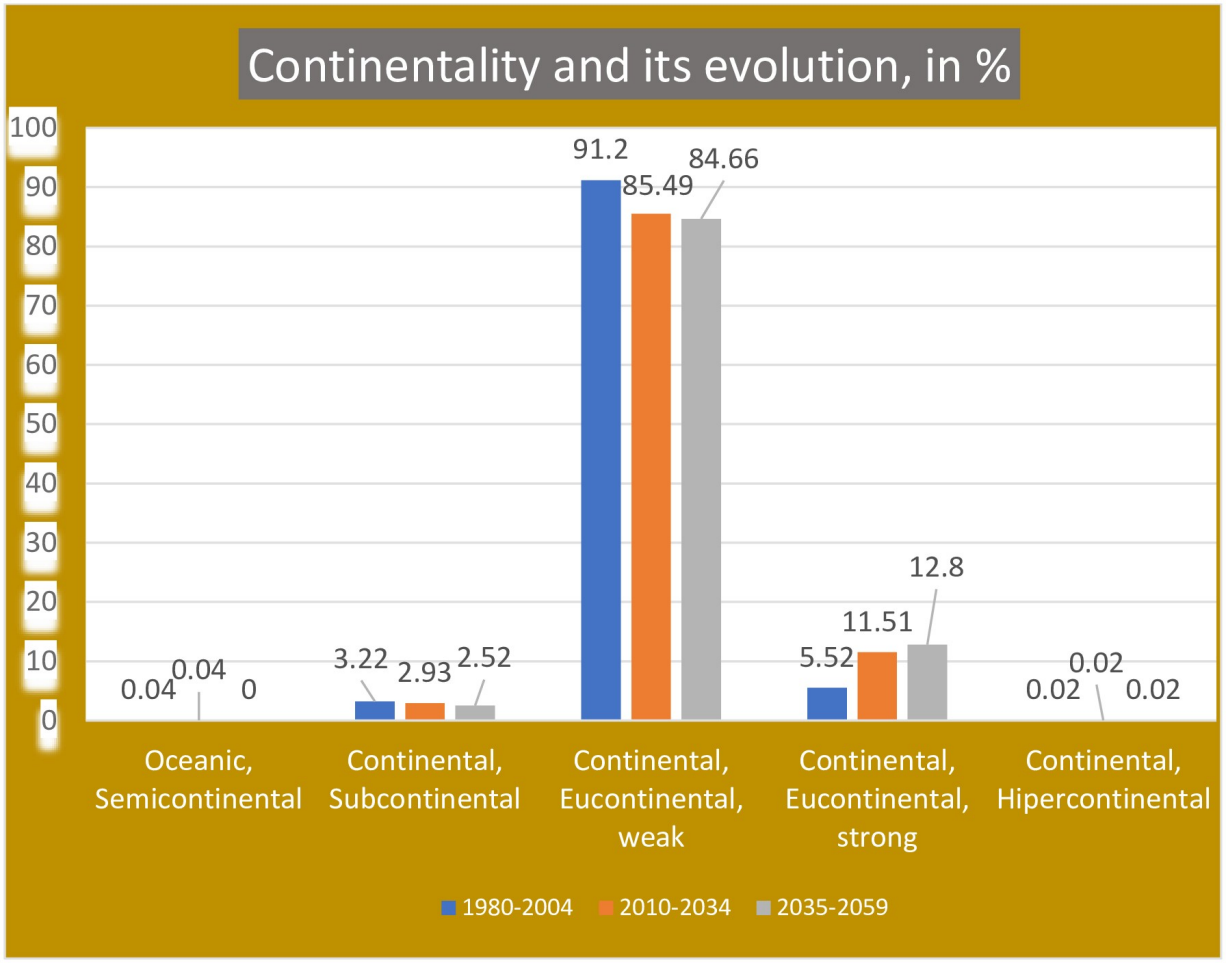
Continentality and its evolution, in %.

**Fig 2:**
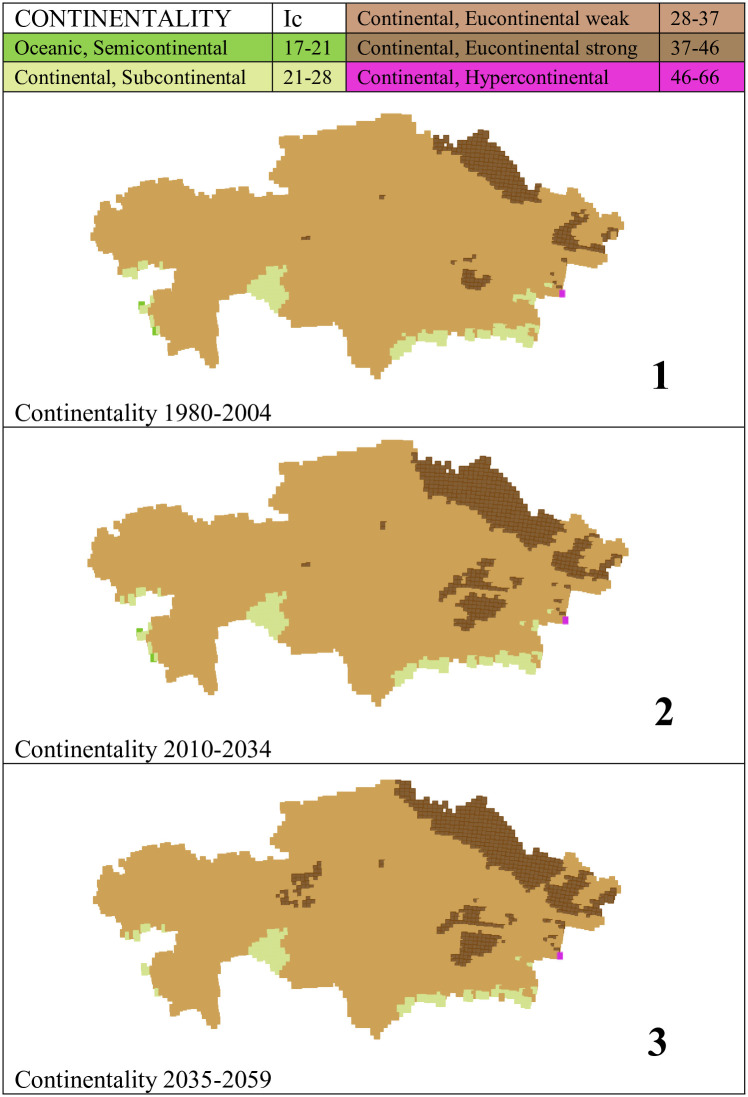
Maps of continentality. (Our own elaboration).

In Kazakhstan, according to the results of the bioclimatic classification shown in S4 Table, we found only the 4 most continental subtypes of the 9 continental subtypes considered in [49]. See Table 2. Of these, the most oceanic subtype and the most continental subtype have minimal representations, limited to one or two points, while the vast majority of the territory, between 96% and 97%, has very high continentality, with continental indexes between 28 and 46. As the eucontinental subtype occupies almost the entire country, we consider two levels, weak and strong, to better appreciate the distribution and evolution of this subtype over time (Table 3, and Figs 1 and 2). That is, almost the entire country has a strong temperature contrast between summer and winter. In the PRECIS projection, this strong continentality is accentuated as the 21^st^ century progresses. The most oceanic areas are the shores of the Caspian Sea, the depression of the Aral Sea, and the SE mountainous flange. The strongest continentality is observed in the NE one-third and the E central part of the country (Fig 2). According to PRECIS projections, from the beginning to the middle of the 21^st^ century, continentality increases in 7.3% of the country.

**Table 3:** Continentality in Kazakhstan.

Ic	CONTINENTALITY	Periods, values and percentages of total territory						Gains/losses in %
		1980–2004		2010–2934		2035 2059		
		Values	%	Values	%	Values	%	
**17–21**	**Oceanic, Semicontinental**	2	**0.04**	2	**0.04**	0	**0.00**	**-0.04**
**21–28**	**Continental, Subcontinental**	147	**3.22**	134	**2.93**	115	**2.52**	**-0.70**
**28–37**	**Continental, Eucontinental, weak**	4167	**91.20**	3906	**85.49**	3868	**84.66**	**-6.54**
**37–46**	**Continental, Eucontinental, strong**	252	**5.52**	526	**11.51**	585	**12.80**	**7.29**
**46–66**	**Continental, Hypercontinental**	1	**0.02**	1	**0.02**	1	**0.02**	**0.00**

### Macrobioclimates

**Fig 3:**
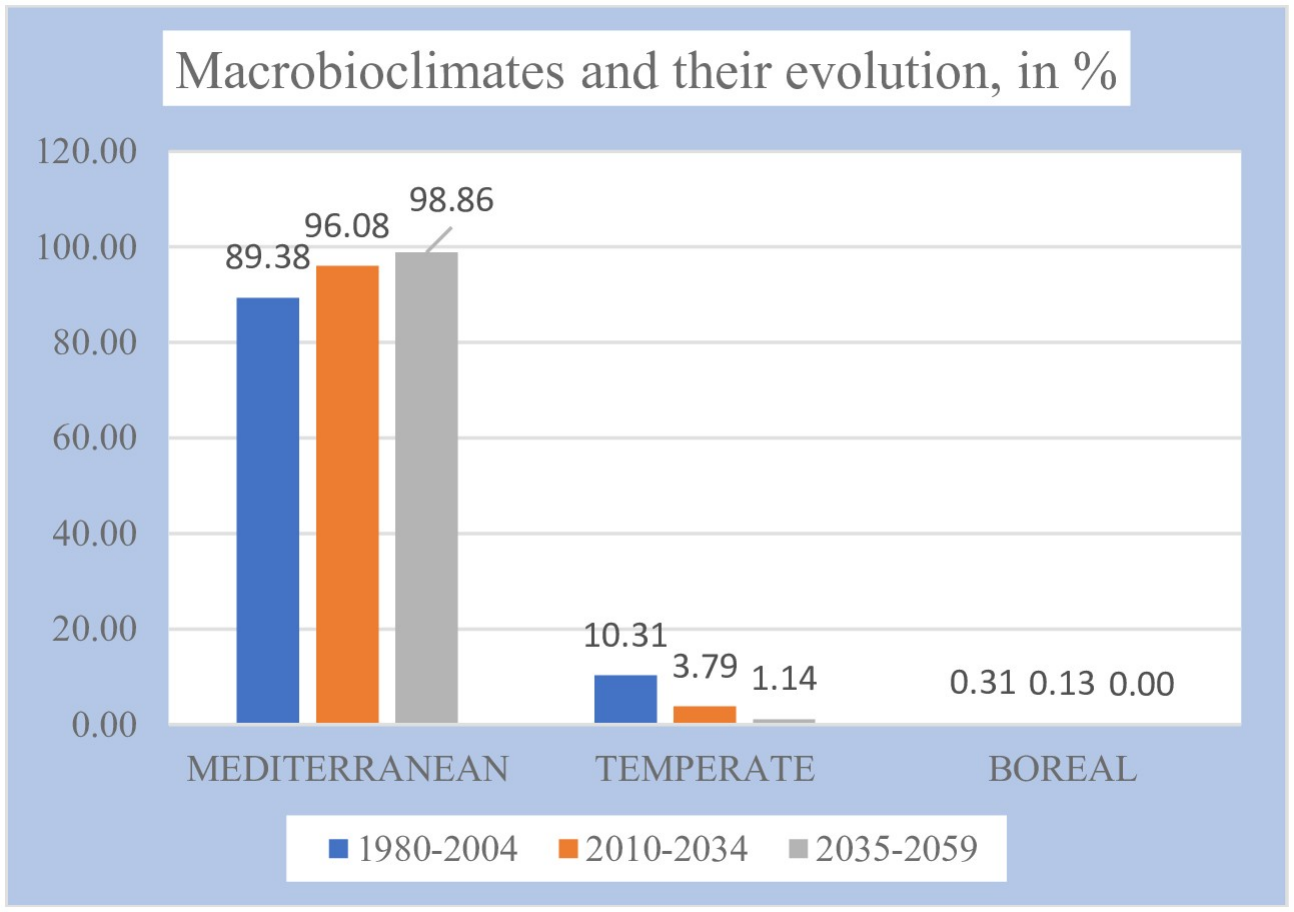
Macrobioclimates and their evolution.

**Fig 4:**
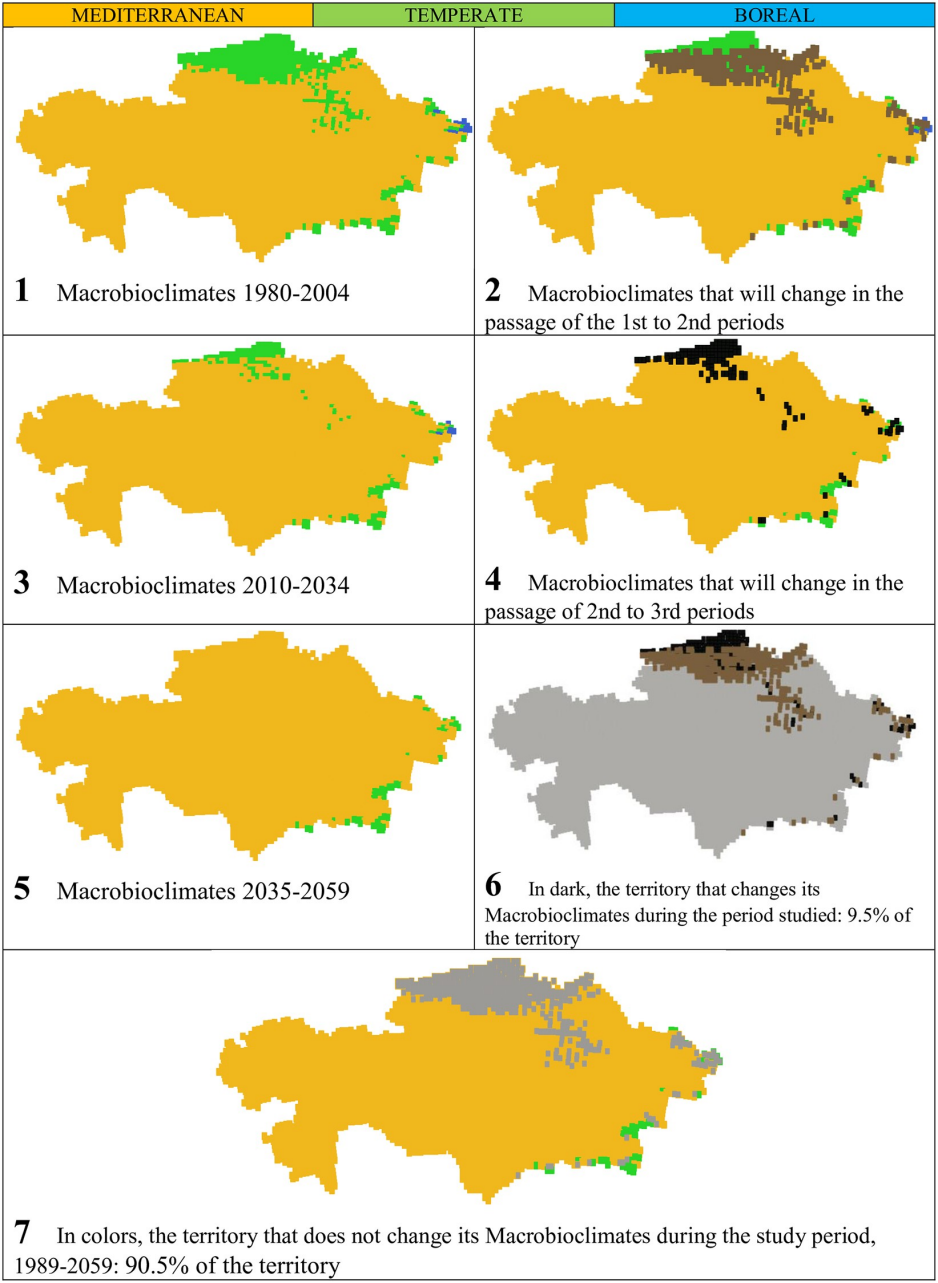
Maps of macrobioclimates and their evolution. (Our own elaboration).

In Table 4 and Figs 3 and 4, it is seen that Kazakhstan is mostly a mediterranean country and that it is becoming almost exclusively mediterranean as the 21^st^ century progresses (Fig 4, maps 1, 3, and 5). The few and small areas of temperate macrobioclimate appear towards the central and northern parts of the country, as well as on its E and SE mountainous edges. The few and small areas of the boreal macrobioclimate appear in the extreme E of the country, in the Altai Massif, next to the China and Russia borders. With the advance of the 21^st^ century, the mediterranean macrobioclimate increases its extent by 9.5% of the territory, while the temperate macrobioclimate decreases in the same proportion, and the boreal macrobioclimate disappears. The mediterranean macrobioclimate pushes the other two macrobioclimates towards the north and towards all the central and peripheral mountains. The change affects only 9.5% of the territory (see Table 4 and Fig 4, map 6).

**Table 4:** Macrobioclimates.

Macrobioclimates	Periods, values and percentages of total territory						Gains/losses in %
	1980–2004		2010–2934		2035 2059		
	Values	%	Values	%	Values	%	
**MEDITERRANEAN**	4084	**89.38**	4390	**96.08**	4517	**98.86**	9.5
**TEMPERATE**	471	**10.31**	173	**3.79**	52	**1.14**	-9.2
**BOREAL**	14	**0.31**	6	**0.13**	0	**0.00**	-0.3

### Bioclimates

**Fig 5:**
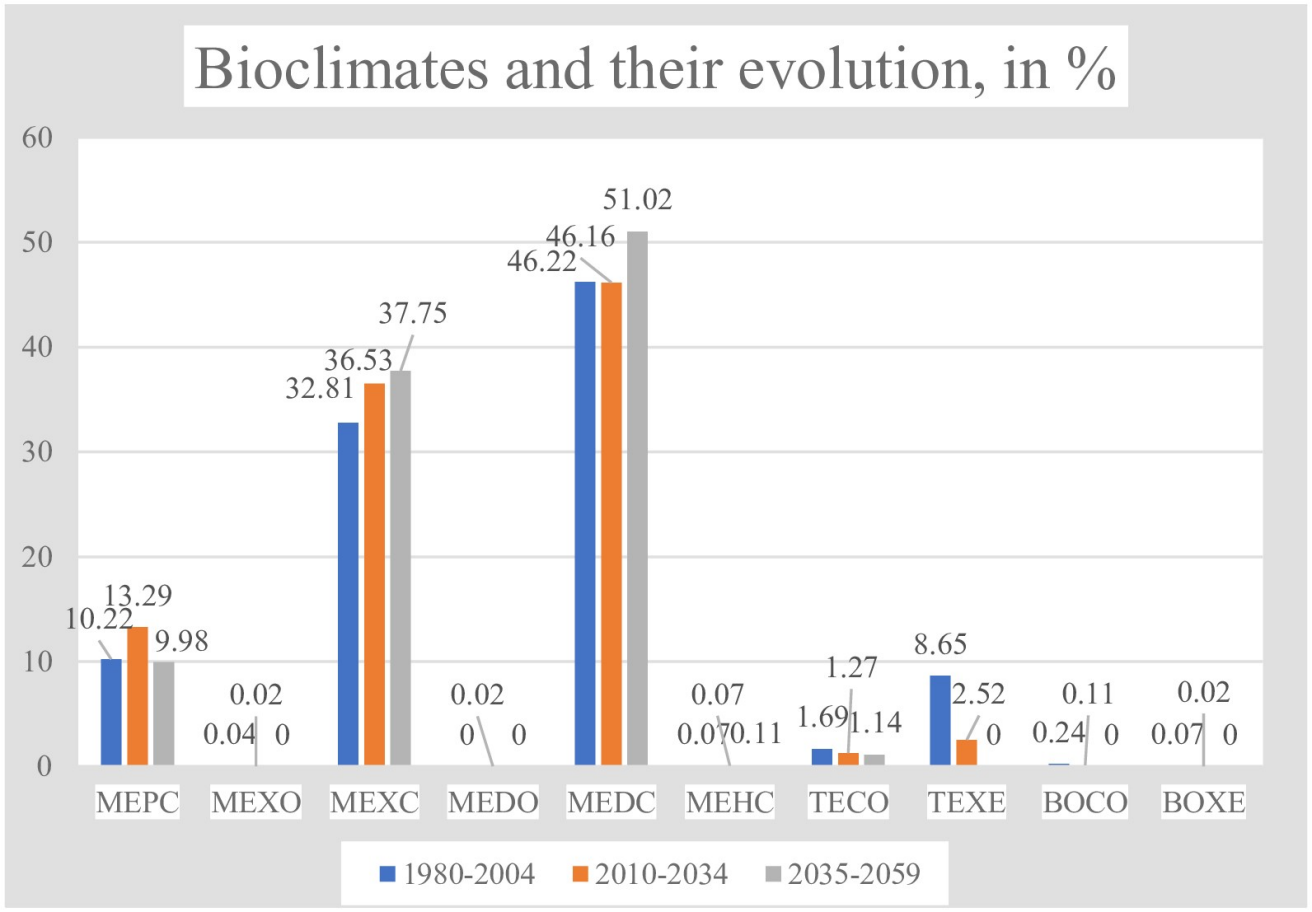
Bioclimates and their evolution.

**Fig 6:**
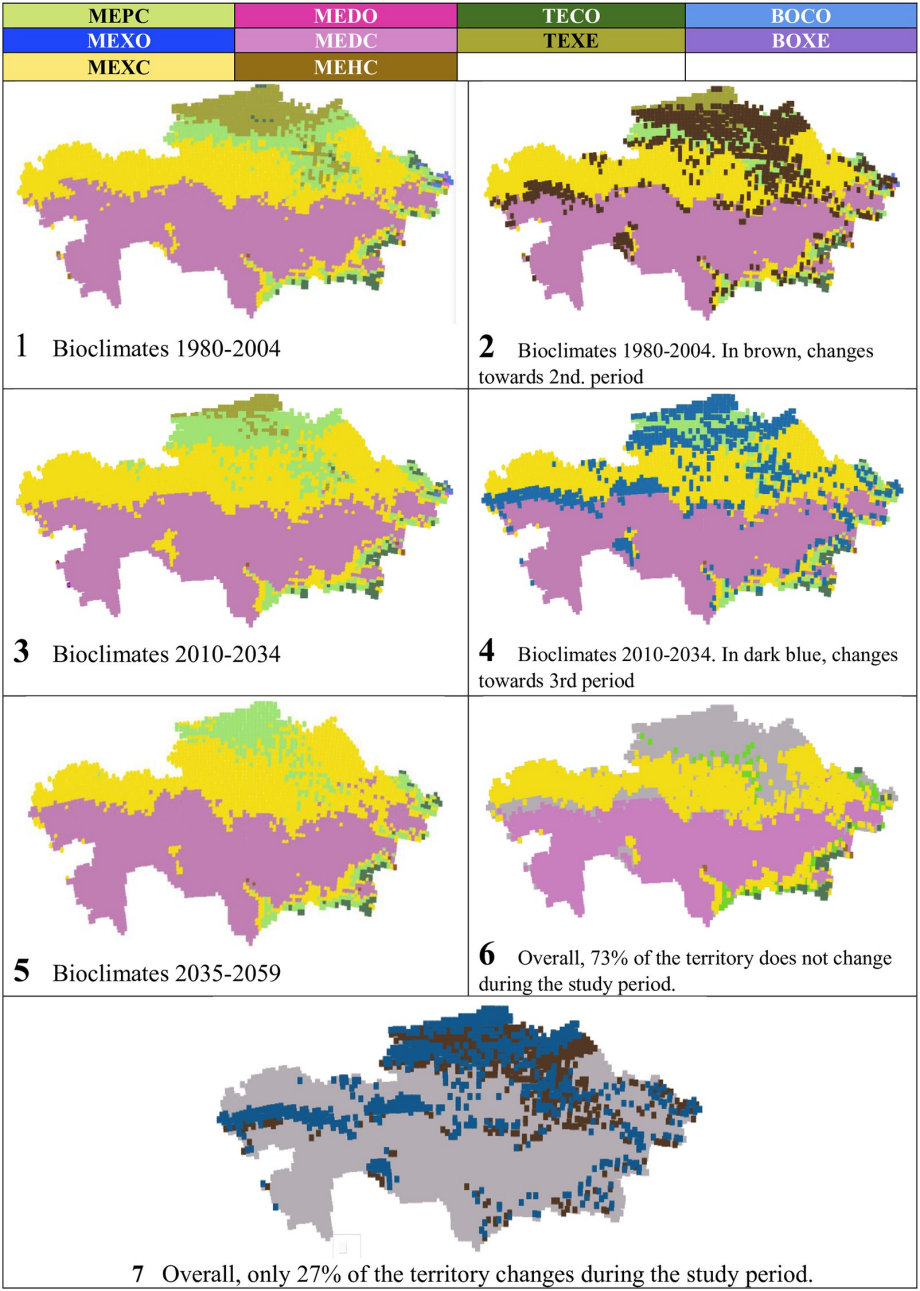
Maps of bioclimates and their evolution. (Our own elaboration).

10 bioclimates were detected in the Kazakhstan territory, according to the projection considered, and for the time intervals studied: 6 mediterranean bioclimates, 2 temperate bioclimates, and 2 boreal bioclimates (see Table 5 and Figs 5 and 6).

**Table 5:** Bioclimates and their evolution.

Bioclimates	Periods, values and percentages of total territory						gains/losses in %
	1980–2004		2010–2034		2035 2059		
	Values	%	Values	%	Values	%	
MEPC	467	10.22	607	13.29	456	9.98	-0.24
MEXO	2	0.04	1	0.02	0	0	-0.04
MEXC	1499	32.81	1669	36.53	1725	37.75	4.95
MEDO	0	0	1	0.02	0	0	0
MEDC	2112	46.22	2109	46.16	2331	51.02	4.79
MEHC	3	0.07	3	0.07	5	0.11	0.02
TECO	77	1.69	58	1.27	52	1.14	-0,53
TEXE	395	8.65	115	2.52	0	0	-8.65
BOCO	11	0.24	5	0.11	0	0	-0.24
BOXE	3	0.07	1	0.02	0	0	-0.07
TOTALS	4569	100	4569	100	4569	100	---
**TOTAL CONTINENTAL BIOCLIMATES**	4169	**91.25**	4451	**97.43**	4569	**100**	**8.75**

The great predominance of the mediterranean macrobioclimate in Kazakhstan, 90–98% of the territory, is distributed unequally among 6 mediterranean bioclimates, MEPC (mediterranean pluviseasonal continental), MEXO (mediterranean xeric oceanic), MEXC (mediterranean xeric continental), MEDO (mediterranean desertic oceanic), MEDC (mediterranean desertic continental) and MEHC (mediterranean hyperdesertic continental). The most extensive bioclimate in the three periods is the MEDC (46–51%), followed by the MEXC (32–27%) and the MEPC (approximately 10%). On the other hand, three mediterranean bioclimates, MEXO, MEDO and MEHC, occupy minimal areas of 5–1 grid cells.

The two temperate bioclimates are also unevenly distributed; TECO (temperate continental), which covers only 1.7% of the territory during the first period, progressively loses some of its representation but is present in all three periods considered. In contrast, TEXE (temperate xeric) begins covering 8.6% of the country, decreases to 2.5% in the second period and disappears completely in the third period.

The two boreal bioclimates, BOCO (boreal continental) and BOXE (boreal xeric), both represented by only tenths or hundredths of % of the total territory, decrease in the second period, and disappear in the third period.

The predominant bioclimates form broad E-W bands (Table 5 and Figs 5 and 6). The most extensive bioclimate in the three periods, MEDC, occupies almost 1/2 of the territory, in the south-central part of the country, except for the E and SE mountains; the next bioclimate in extent, MEXC, covering 1/3 of the territory, occupies the north-central half of the country and borders the MEDC widely on the N but also on the S; the next bioclimate, MEPC, with 10% of the territory, borders the MEXC, both on the N and on the S; and, finally, all of this ordered set of bioclimates appears bordered to the N, E and SE by the temperate bioclimates, TEXE to the N, and TECO in the NE, E and SE mountains. In turn, BOCO and BOXE only have very small representation in the Altai massif.

According to PRECIS projections, from the beginning to the middle of the 21^st^ century, the climatic change in Kazakhstan will cause 9.8% of the country’s surface to change its bioclimate. The bioclimates MEDC and MEXC increase their representation, mainly at the expense of TEXE and in a very small percentage, of TECO, BOCO and BOXE (Table 5 and Figs 5 and 6). As seen in Fig 6, map 7, changes in bioclimates occur almost exclusively in the northern half of Kazakhstan, and such changes are mainly located in the contact areas between bioclimates (Fig 6, maps 2 and 4).

In the last row of Table 5, the territories of the continental bioclimates of each of the three periods have been added, both in values and in %; there is a progressive increase in continental bioclimates, which reach 100% in the third period. That is, Kazakhstan, which already begins the 21^st^ century with a very high proportion of continental bioclimates, 91.25%, changes, towards the middle of the century, to be 100% occupied by continental bioclimates.

### Bioclimatic variants

**Fig 7:**
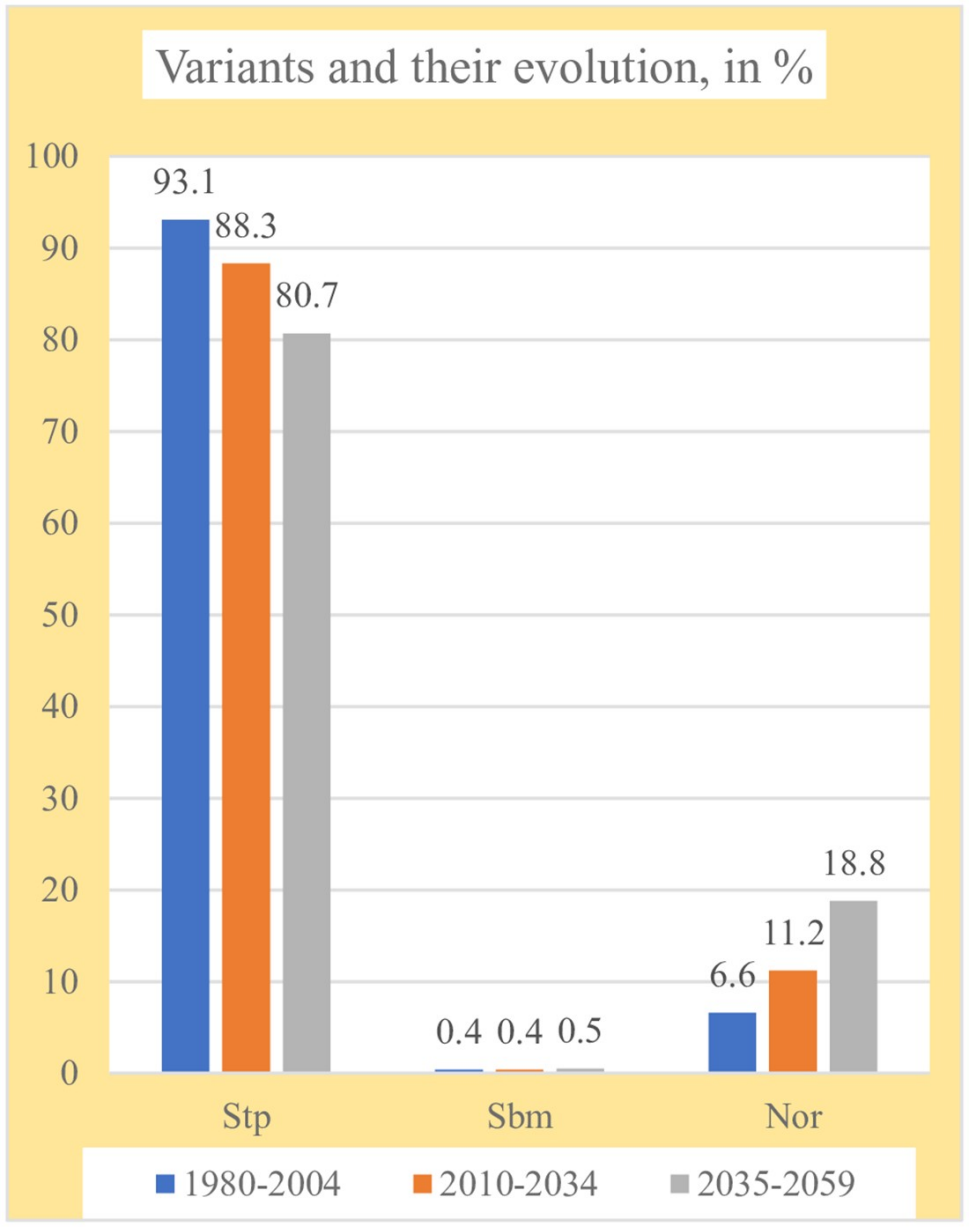
Variants and their evolution.

**Fig 8:**
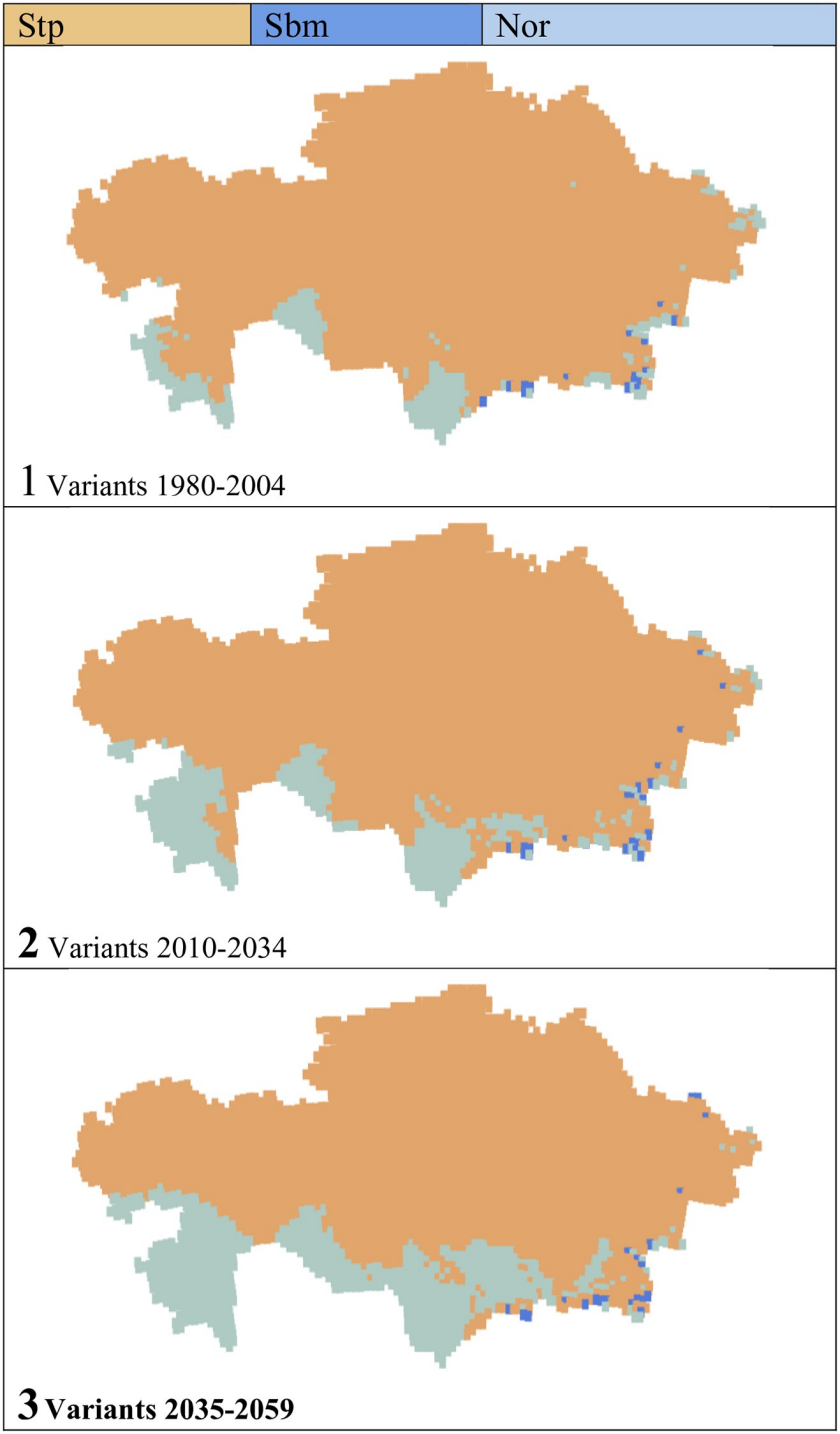
Maps of variants and their evolution. (Our own elaboration).

With the PRECIS prediction used data, three bioclimatic variants have been detected in Kazakhstan: steppe, submediterranean and normal (Table 6, Figs 7 and 8). The steppe variant predominates in all three periods, occupying 93 to 80% of the territory, because, as the century progresses, it loses 12.4% of its extent. The submediterranean variant occupies very little area, only 0.4–0.5% of the territory and appears exclusively in the E and SE mountainous regions. Finally, the normal variant occupies from 6 to 18% of the surface and is mainly found in the S, SE and E parts of the country. The normal variant gains 12.4% from the first to the third period. The normal variant increases at the expense of the steppe variant; that is, with the advance of the 21^st^ century, the steppe variant disappears in 12.4% of Kazakhstan.

**Table 6:** Bioclimatic variants.

Bioclimatic variants	Periods, values and percentages of total territory						Gains/losses in %
	1980–2004		2010–2034		2035 2059		
	Values	%	Values	%	Values	%	
Stp	4252	93.1	4035	88.3	3687	80.7	-12.4
Sbm	16	0.4	20	0.4	21	0.5	0.1
Nor	301	6.6	514	11.2	861	18.8	12.3

### Thermotypes

**Fig 9:**
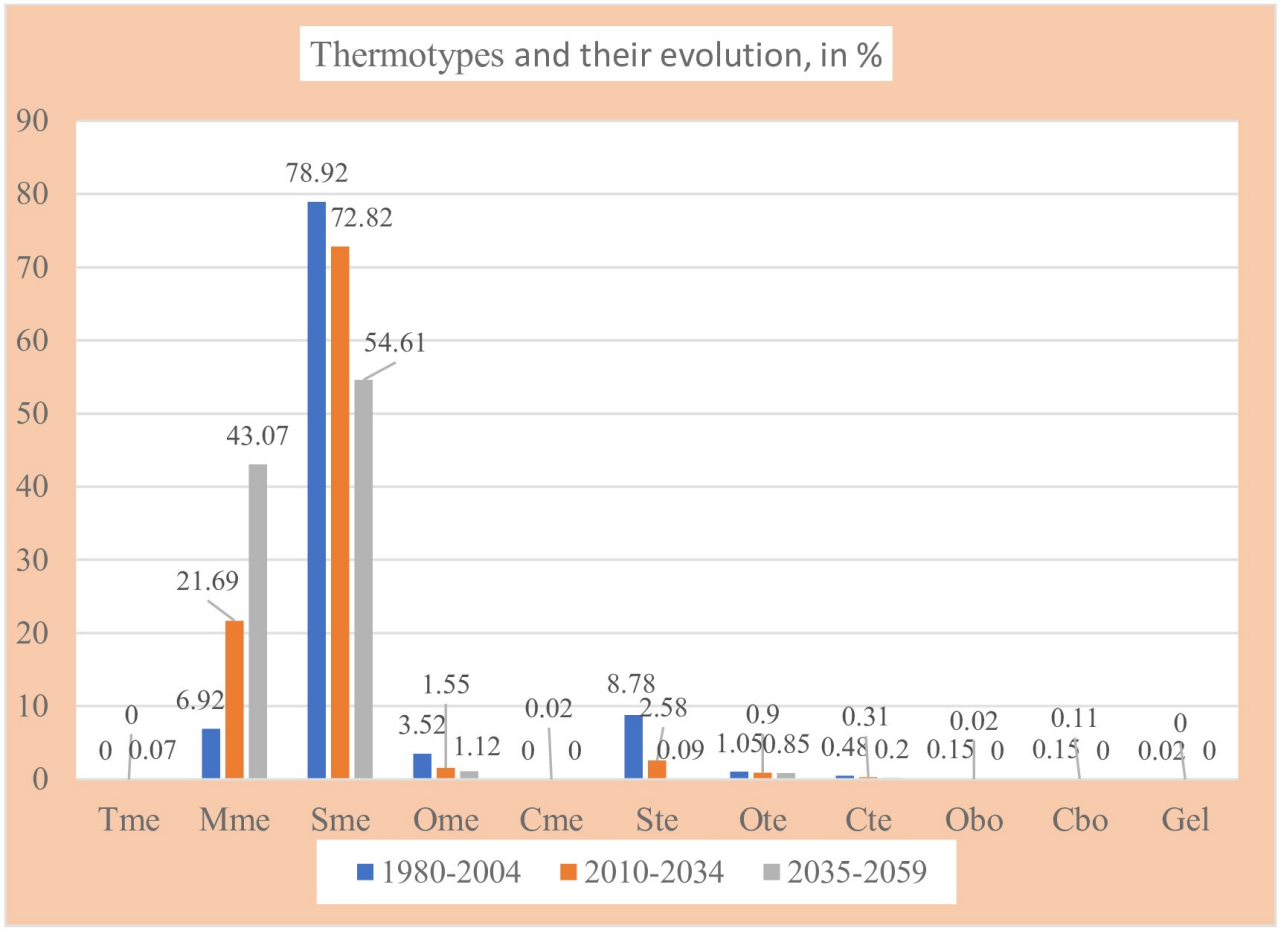
Thermotypes and their evolution.

**Fig 10:**
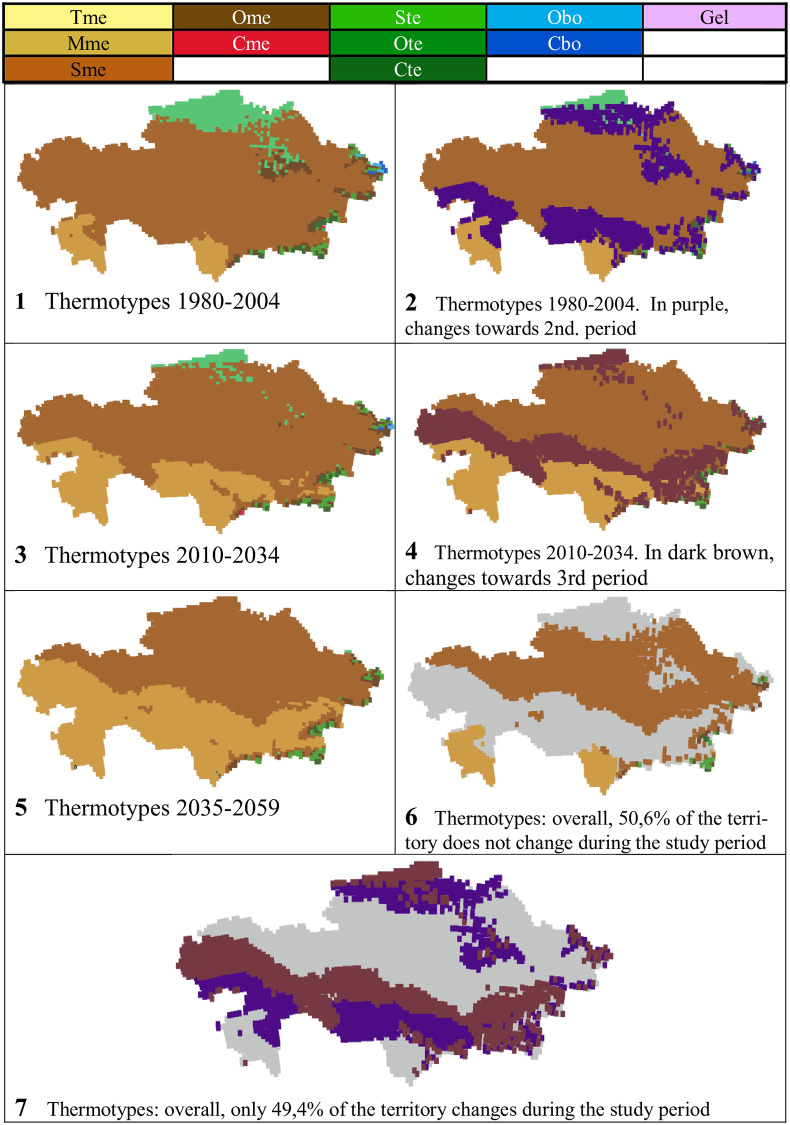
Maps of thermotypes and their evolution. (Our own elaboration).

On the whole, from the 1980–2059 PRECIS projections in Kazakhstan, we found representation of 11 thermotypes: 5 mediterranean, 3 temperate, 2 boreal and 1 gelid (Table 7 and Figs 9 and 10).

**Table 7:** Thermotypes and their evolution.

Thermotypes	Periods, values and percentages of total territory						gains/losses in %
	1980–2004		2010–2034		2035 2059		
	Points	%	Points	%	Points	%	
Tme	0	0	0	0	3	0.07	0.07
Mme	316	6.92	991	21.69	1968	43.07	36.16
Sme	3606	78.92	3327	72.82	2495	54.61	-24.32
Ome	161	3.52	71	1.55	51	1.12	-2.41
Cme	0	0	1	0.02	0	0	0
Ste	401	8.78	118	2.58	4	0.09	-8.69
Ote	48	1.05	41	0.9	39	0.85	-0.2
Cte	22	0.48	14	0.31	9	0.2	-0.28
Obo	7	0.15	1	0.02	0	0	-0.15
Cbo	7	0.15	5	0.11	0	0	-0.15
Gel	1	0.02	0	0	0	0	-0.02

In an overwhelmingly mediterranean territory, it is natural that the best-represented thermotypes were the mediterranean ones: only Sme (supramediterranean thermotype) and Mme (mesomediterranean thermotype) together cover between 86% and 98% of the territory, according to the different periods. Regarding the temperate thermotypes, the best represented correspond to Ste (supratemperate thermotype) in the first and second periods, followed by Ote (orotemperate thermotype).

Regarding the distribution of the thermotypes in the territory (see Fig 10, maps 1, 3 and 5, corresponding to the 1st, 2nd and 3rd periods, respectively) it can be observed that Sme, the most extensive thermotype in all the periods, forms a very large continuous latitudinal band from E to W, which moves N, pushed by Mme; Mme, located in the S central and SW parts of the country during the first period, increases its area to the N, to occupy almost half of the country; Tme (thermomediterranean thermotype) only appears in the third period and occupies three separate points along the S border of the country; Ome (oromediterranean thermotype) is found on some slopes of the mountain systems; and Cme (cryoromediterranean thermotype) only occupies one point in the second period on the S border of the country. Regarding the temperate and boreal thermotypes, Ste is located in the N central Kazakhstan; Ote (orotemperate thermotype) and Cte (cryorotemperate thermotype) occupy the slopes of the mountain systems in the NE, E and SE parts of the country; Obo (oroboreal thermotype) and Cbo (cryoroboreal thermotype) only appear at the far NE end of the country; and the gelid thermotype only appears on the eastern border with China.

In general, all the supra-, oro-, cryoro- and gel- thermotypes progressively lose ground as the 21^st^ century progresses: only the meso- and thermo- thermotypes increase their areas. There is thus a clear tendency to increase thermicity. In this sense, the progressive increase in Mme is very striking, accompanied by the progressive decrease in Sme (Table 7). According to PRECIS projections, from the beginning to the middle of the 21^st^ century, climate change in Kazakhstan will cause 36.2% of the country to change its thermotype (Table 7 and Fig 10, maps 6 and 7).

### Ombrotypes

**Fig 11:**
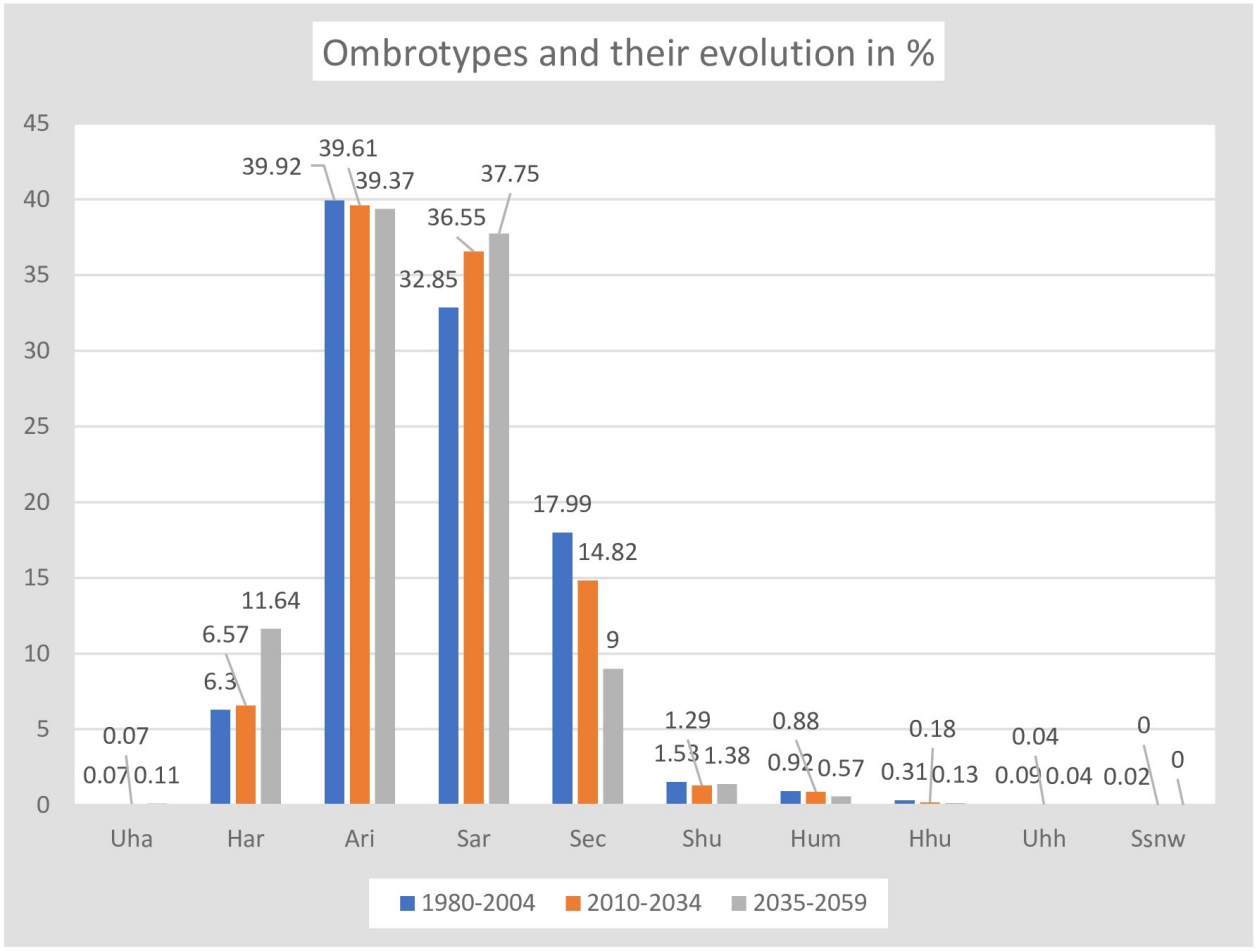
Ombrotypes and their evolution.

**Fig 12:**
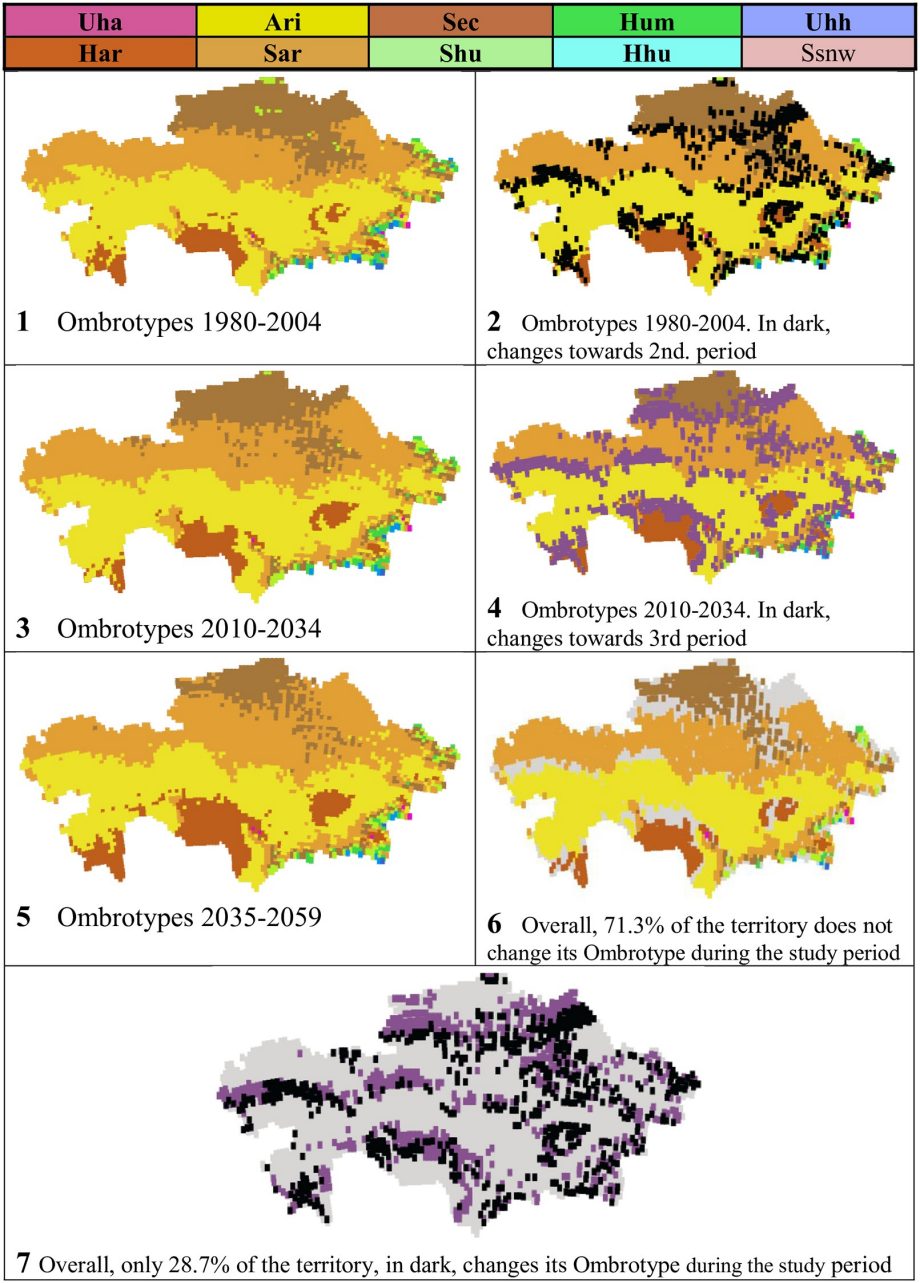
Maps of ombrotypes and their evolution. (Our own elaboration).

In Kazakhstan, according to PRECIS projections, 10 ombrotypes were found: 9 from the Uha (ultrahyperarid), Har (hyperarid), Ari (arid), Sec (dry), Shu (subhumid), Hum (humid), Hhu (hyperhumid) and Uhh (ultrahyperhumid), as well as Ssnw (supersnowy) (Table 8, and Figs 11 and 12). However, the 5 best-represented ombrotypes already cover 98% of the territory in each of the considered periods. The two best represented ombrotypes are Ari, which covers almost 40% of the territory, followed by Sar with approximately 36% (Figs 11 and 12).

**Table 8:** Ombrotypes and their evolution.

OMBROTYPES	Periods, values and percentages of total territory						Gains/losses in %
	1980–2004		2010–2034		2035–2059		
	Points	%	Points	%	Points	%	
**Uha**	3	0.07	3	0.07	5	0.11	0.04
**Har**	288	6.30	300	6.57	532	11.64	5.34
**Ari**	1824	39.92	1810	39.61	1799	39.37	-0.55
**Sar**	1501	32.85	1670	36.55	1725	37.75	4.90
**Sec**	822	17.99	677	14.82	411	9.00	-9.00
**Shu**	70	1.53	59	1.29	63	1.38	-0.15
**Hum**	42	0.92	40	0.88	26	0.57	-0.35
**Hhu**	14	0.31	8	0.18	6	0.13	-0.18
**Uhh**	4	0.09	2	0.04	2	0.04	-0.04
**Ssnw**	1	0.02	0	0.0	0	0.0	-0.02

Aside from ombrotype Uha, which only occupies three-five points isolated from each other in the ESE, the ombrotypes Har, Ari, Sar and Sec tend to form continuous latitudinal bands from E to W and in succession S to N: that is, there is a latitudinal sequence towards more humid ombrotypes towards the N. In turn, the mountains of the central and peripheral NE, E and SE regions have an altitudinal sequence of ombrotypes, increasingly wet as the altitude increases.

Among the three time periods considered, there is a strong decrease in Sec and all the wet ombrotypes in favor of the arid ombrotypes: Sar and Har increase, while Ari retains its representation. That is, a strong tendency towards the aridification of the territory is noted. During the studied period, from 1980 to 2059, the climatic change in Kazakhstan causes 10.3% of the country to change its ombrotype (Table 8). The changes are mainly located in the contacts between the areas of the ombrotypes (Fig 12, maps 6 and 7).

### Isobioclimates

The unique combinations of a bioclimate, a thermotype and an ombrotype are known as isobioclimates. An isobioclimate is a bioclimatic space that plants feel as homogeneous: it is a natural phytotron, whose walls or limits are the upper and lower thresholds of each of its component bioclimatic units. We assigned the corresponding isobioclimate to each of our 4569 grid cells in each of the three periods. The results are given in S4 Table.

Table 9 “Isobioclimates and their evolution” brings together all the isobioclimates detected in the three periods considered, as well as their coverage, expressed both in grid cells and in %. In the last column, the differences in coverage between the third and the first period are expressed, that is, the increase or decrease in the isobioclimate area with the progress of the 21^st^ century. The yellow shaded cells indicate the isobioclimates that increase their representation over time; the cells shaded in gray, the isobioclimates that either do not change, or that diminish their presence with the passage of time. Percent values equal to 0 are shaded in pink. Isobioclimates, whose order number (left column) appears shaded in gray, are the best represented in Kazakhstan.

**Table 9:** Isobioclimates and their evolution.

Order No.	Isobioclimates			1980–2004		2010–2034		2035–2059		Difference in % 2035–1980
				Grid cells	%	Grid cells	%	Grid cells	%	
1	MEPC	Mme	Sec	0	0.00	4	0.09	10	0.22	0.2
2	MEPC	Mme	Shu	0	0.00	0	0.00	1	0.02	0.0
**3**	MEPC	Sme	Sec	348	7.62	531	11.62	383	8.38	0.8
4	MEPC	Sme	Shu	9	0.20	11	0.24	18	0.39	0.2
**5**	MEPC	Ome	Sec	87	1.90	28	0.61	18	0.39	-1.5
6	MEPC	Ome	Shu	20	0.44	30	0,. 6	24	0.53	0.1
7	MEPC	Ome	Hum	3	0.07	2	0.04	2	0.04	-0.02
8	MEPC	Cme	Shu	0	0.00	1	0.02	0	0.00	0.00
9	MEXO	Mme	Sar	1	0.02	1	0.02	0	0.00	-0.02
10	MEXO	Sme	Sar	1	0.02	0	0.00	0	0.00	-0.02
**11**	MEXC	Mme	Sar	17	0.37	46	1.01	224	4.90	4.5
**12**	MEXC	Sme	Sar	1431	31.32	1612	35.28	1494	32.70	1.4
13	MEXC	Ome	Sar	51	1.12	11	0.24	7	0.15	-1.0
14	MEDO	Mme	Ari	0	0.00	1	0.02	0	0.00	0.00
15	MEDC	Tme	Ari	0	0.00	0	0.00	2	0.04	0.04
16	MEDC	Tme	Har	0	0.00	0	0.00	1	0.02	0.02
**17**	MEDC	Mme	Ari	197	4.31	718	15.71	1237	27.07	22.8
**18**	MEDC	Mme	Har	101	2.21	220	4.82	492	10.77	8.6
**19**	MEDC	Sme	Ari	1627	35.61	1091	23.88	560	12.26	-23.4
**20**	MEDC	Sme	Har	187	4.09	80	1.75	39	0.85	-3.2
21	MEHC	Mme	Uha	0	0.00	1	0.02	4	0.09	0.1
22	MEHC	Sme	Uha	3	0.07	2	0.04	1	0.02	-0.04
23	TECO	Ste	Shu	11	0.24	2	0.04	2	0.04	-0.2
24	TECO	Ste	Hum	0	0.00	2	0.04	2	0.04	0.04
**25**	TECO	Ote	Shu	19	0.42	10	0,.2	18	0.39	0.0
**26**	TECO	Ote	Hum	21	0.46	28	0.61	17	0.37	-0.1
27	TECO	Ote	Hhu	3	0.07	2	0,.4	4	0.09	0.0
28	TECO	Cte	Shu	0	0.00	0	0.00	1	0.02	0.02
29	TECO	Cte	Hum	9	0.20	6	0.13	4	0.09	-0.1
30	TECO	Cte	Hhu	9	0.20	6	0.13	2	0.04	-0.2
31	TECO	Cte	Uhh	4	0.09	2	0.04	2	0.04	-0.04
32	TECO	**Gel**	SSn	1	0.02	0	0.00	0	0.00	-0.02
**33**	TEXE	Ste	Sec	381	8.34	112	2.45	0	0.00	-8.3
34	TEXE	Ste	Shu	9	0.20	2	0.04	0	0.00	-0.2
35	TEXE	Ote	Sec	4	0.09	1	0.02	0	0.00	-0.1
36	TEXE	Ote	Shu	1	0.02	0	0.00	0	0.00	-0.02
37	BOCO	Obo	Shu	0	0.00	1	0.02	0	0.00	0.00
38	BOCO	Obo	Hum	4	0,.9	0	0.00	0	0.00	-0.1
39	BOCO	Cbo	Shu	0	0.00	2	0.04	0	0.00	0.00
40	BOCO	Cbo	Hum	5	0,.1	2	0.04	0	0.00	-0.1
41	BOCO	Cbo	Hhu	2	0.04	0	0.00	0	0.00	-0.04
42	BOXE	Obo	Sec	2	0.04	1	0,.02	0	0.00	-0.04
43	BOXE	Obo	Shu	1	0.02	0	0.00	0	0.00	-0.02

As seen in Table 9, in the whole of Kazakhstan and for the three periods considered, 43 isobioclimates—22 mediterranean, 14 temperate, and 7 boreal—were detected, with very unequal representation. Among them, 11 isobioclimates (whose order numbers are bold and shaded in gray in the table) cover 96 to 98% of the territory according to the periods. The remaining 32 isobioclimates occupy the remaining 4–2% of the territory; that is, they have very small territorial representations.

**Fig 13:**
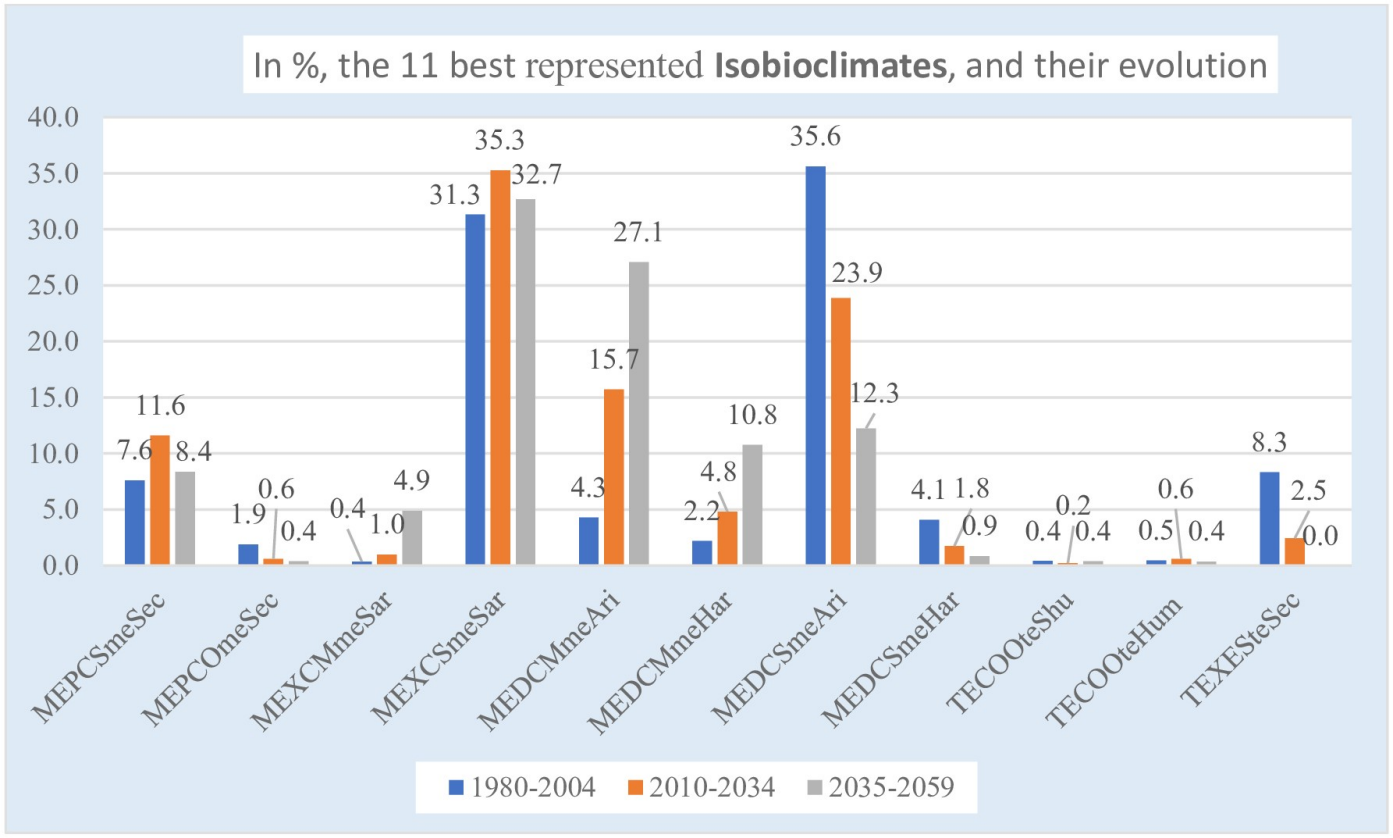
The 11 best represented isobioclimates and their evolution.

**Fig 14:**
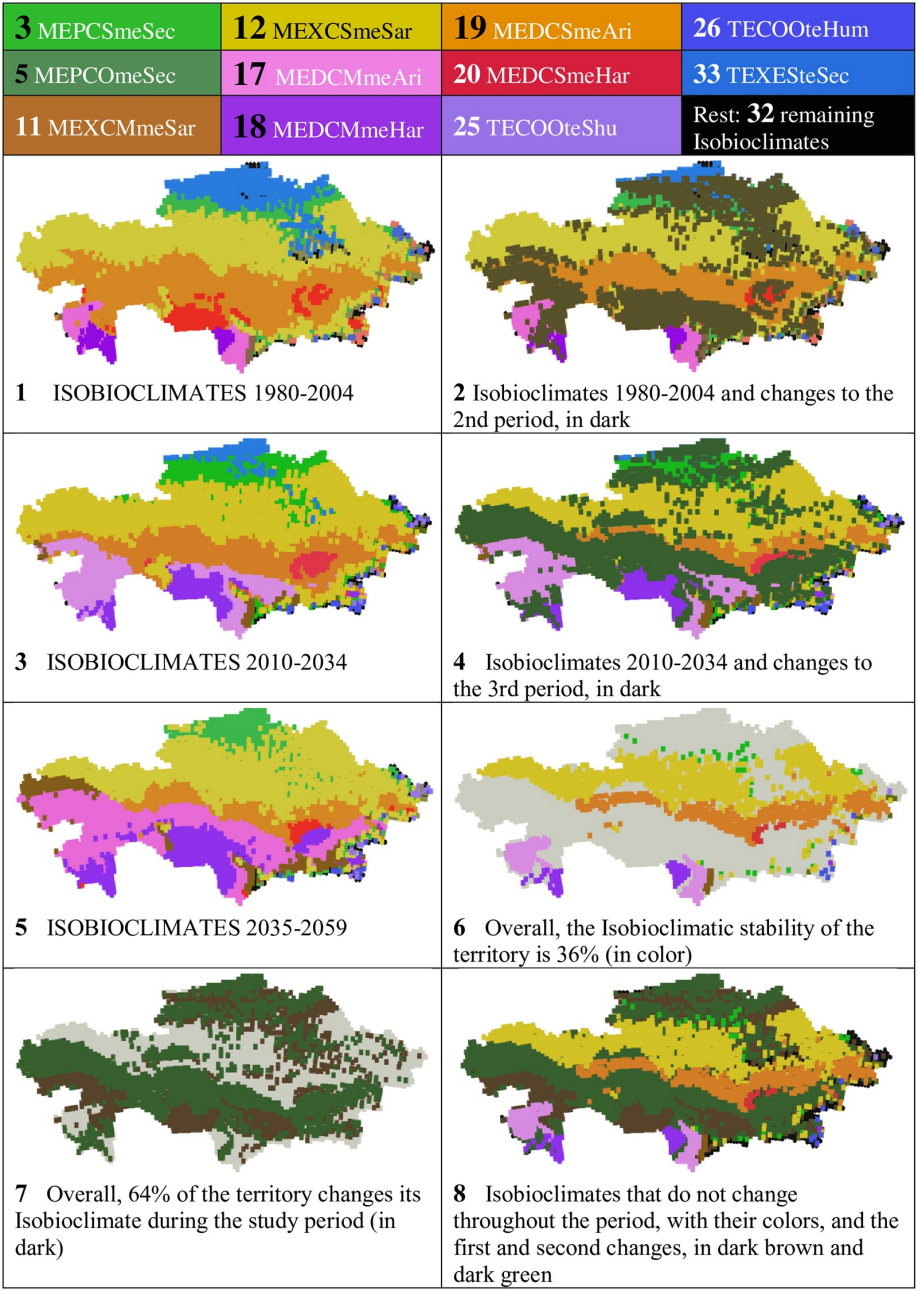
Maps of the 11 best represented isobioclimates and their evolution. (Our own elaboration).

In Table 10 and in Figs 13 and 14, we have gathered the information about the 11 best represented isobioclimates, with their % of area according to the periods, the % of gain/loss between the first and third periods, and their geographical distribution in the three study periods. As can be seen, among the great losses or gains, those that lose are Sme or TEXE, and those that gain are all Mme.

**Table 10:** Best represented isobioclimates and their evolution.

	Best represented Isobioclimates	1980	2010	2035	Gains/losses, in %
		%	%	%	
**3**	MEPC Sme Sec	7.6	11.6	8.4	0.8
**5**	MEPC Ome Sec	1.9	0.6	0.4	-1.5
**11**	MEXC Mme Sar	0.4	1.0	4.9	**4.5**
**12**	MEXC Sme Sar	31.3	35.3	32.7	1.4
**17**	MEDC Mme Ari	4.3	15.7	27.1	**22.8**
**18**	MEDC Mme Har	2.2	4.8	10.8	**8.6**
**19**	MEDC Sme Ari	35.6	23.9	12.3	**-23.4**
**20**	MEDC Sme Har	4.1	1.8	0.9	**-3.2**
**25**	TECO Ote Shu	0.4	0.2	0.4	0.0
**26**	TECO Ote Hum	0.5	0.6	0.4	-0.1
**33**	TEXE Ste Sec	8.3	2.5	0.0	**-8.3**
	TOTALS	96.7	98.0	98.1	

Fig 14 is the colored spatial representation of the 11 most extensive isobioclimates in the three considered time periods (maps 1, 3 and 5) (Table 10). In addition, the locations of all the remaining 32 isobioclimates with minimal territories are represented in black: they occupy border positions in the S, SE, E and N parts of the country, with some representation in the Karaganda massif. Further, in Fig 14, maps 2 and 4, we have represented the changes in the geographic distribution of isobioclimates from the 1st to 2nd and from the 2nd to 3rd periods; the most striking change occurs between isobioclimate 17—MEDC Mme Ari, which gains 22.8%, and isobioclimate 19—MEDC Sme Ari, which loses 23.4% (Table 10 and Fig 13): Mme gains, and Sme loses. Actually, all Mme gain, while losses occur for Oro and Supra. In addition, in Fig 14, map 6 shows the territory that does not change its isobioclimate between 1980 and 2059, map 7 shows all the territory that changes its isobioclimate, and map 8 combines the information from maps 6 and 7.

## Discussion

From a purely climatic point of view, some studies have been carried out on projected changes in the temperature and precipitation climatology of Central Asia. Thus, in 2009, the regional scenarios derived from four models among twenty-three AOGCMs (Atmosphere Ocean Global Climate Models) were analyzed [69]. Both annual and seasonal temperature and precipitation scenarios, from HadCM3, CSIRO-Mk3, ECHAM5, and CGCM3, under different IPCC SRES policy scenarios, were studied: all four models demonstrate a good level of sensitivity for arid and semi-arid Central Asia. In their conclusions, pp. 975, the authors affirm: “Central Asia is projected to become warmer during the coming decades. Aridity is expected to increase across the entire region, especially in the western parts of Turkmenistan, Uzbekistan, and Kazakhstan. … The temperature increases are projected to be particularly high in summer and fall, but lower in winter. Significant decrease in precipitation is projected for summer and fall”.

Likewise, in 2017, the projected changes in temperature and precipitation climatology of Central Asia CORDEX Region 8 were studied [70]. Working with the Regional Climate Model 4.3.5, RegCM4.3.5, of the International Center for Theoretical Physics (ICTP), driven by two different CMIP5 global climate models, the HadGEM2-ES and the MPI-ESM-MR, the authors found that general warming and decreases in precipitation for the domain were projected by all models. They also point to relatively high warming in the warm season.

In the projections of the regional model PRECIS [65–66], for the years 1980–2059, we have found those same tendencies of climate change, which we express, quantify and locate cartographically, by applying the bioclimatic method—WBCS of Rivas-Martinez [49]-.

### Increase in thermicity

The forecasts of generalized and progressive climate warming as the 21st century progresses [69, 70] are bioclimatically reflected in our results: 36.2% increase in the area occupied by the warm thermotypes thermo- and meso-, accompanied by 36.2% decrease in the area occupied by all cold thermotypes, supra-, oro-, cryoro- and gel-. See Table 11. In Fig 10—the thermotype maps-, the distribution of the found thermotypes, and their variation in size and position with the passage from one study period to the next, can be seen.

**Table 11:** Gains / losses in warm / cold thermotypes.

Thermobelt	Gains/losses	Change
Thermo	0.07	**Increase in %** **36.2**
Meso	36.16	
Supra	-33.0	**Decrease in %** **-36.2**
Oro	-2.8	
Cryoro	-0.4	
Gel	-0.02	

### Increase in continentality

Both [69, 70] find that the temperature increases are projected to be particularly high in summer. In bioclimatic terms, this means that continentality will increase throughout the region, because as summer temperatures increase more than winter temperatures, the extremes of the climate increase. This is precisely what we have verified in our study: between the end of the 20th century and the middle of the 21st century, 7.3% of the territory of Kazakjistan increases its continentality. (See Figs 1 and 2).

### Increase in annual aridity

In studies on projected climatic change [69, 70] during the end of the 20th century and the first half of the century, both a general warming and decreases in precipitation have been found. That implies increased aridity. This is what we have found when bioclimatically assessing annual aridity using the ombrothermic index, whose intervals determine the ombrotypes. Table 8 shows that only the arid Ombrotypes,–semiarid/Sar, hyperarid/Har and ultrahyperarid/Uha-, increase their area by 10.3% in Kazakhstan, while all the ombrotypes from dry/Sec to subhumid/Shu, humid/Hum, hyperhumid/Hhu, ultrahyperhumid/Uhh and snowy/Ssnw, decrease their area in the territory, in the same proportion. Fig 12 shows the location and extent of the ombrotypes in the three periods of time considered, as well as the changes between periods.

### Increase in summer aridity / Increase in mediterraneity

According to several regional scenarios [69], the temperature increases are projected to be particularly high in summer and fall, together with significant decrease in precipitation also for summer and fall. Working with another Regional Climate Model [70], the same predictions were found: a particularly high increase in temperatures in summer and autumn, along with a decrease in rainfall in the same period. Using the PRECIS predictions, we have also found the same results, which, bioclimatically interpreted, suppose an increase in summer aridity, that is, an increase in mediterraneity. And that is precisely what we have found in our study: as the 21st century progresses, the mediterranean macrobioclimate, characterized by summer aridity, increases in 9.5% of the territory, compared to the same setback of the combined temperate and boreal macrobioclimates, both lacking summer aridity. That is to say, summer aridity, mediterraneity, increases in 9.5% of the territory, throughout the first half of the 21st century, according to PRECIS. (See Table 4 and Figs 3 and 4).

### The mountains role

**Fig 15:**
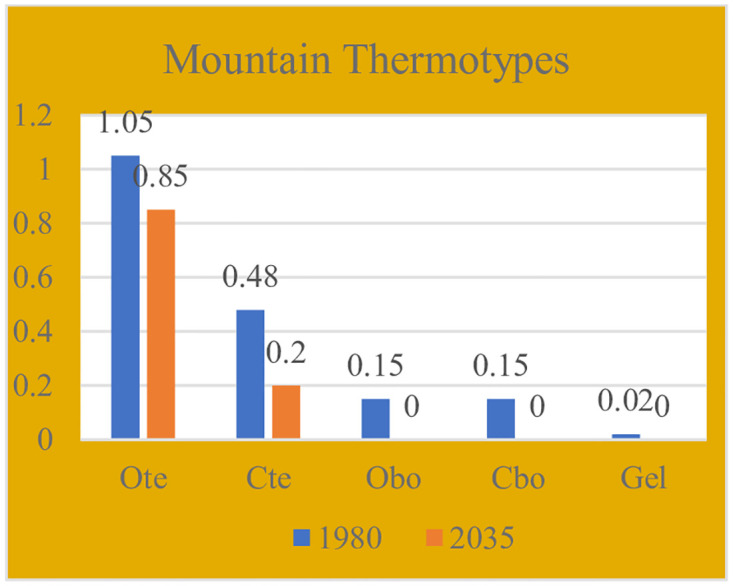
Mountain thermotypes.

**Fig 16:**
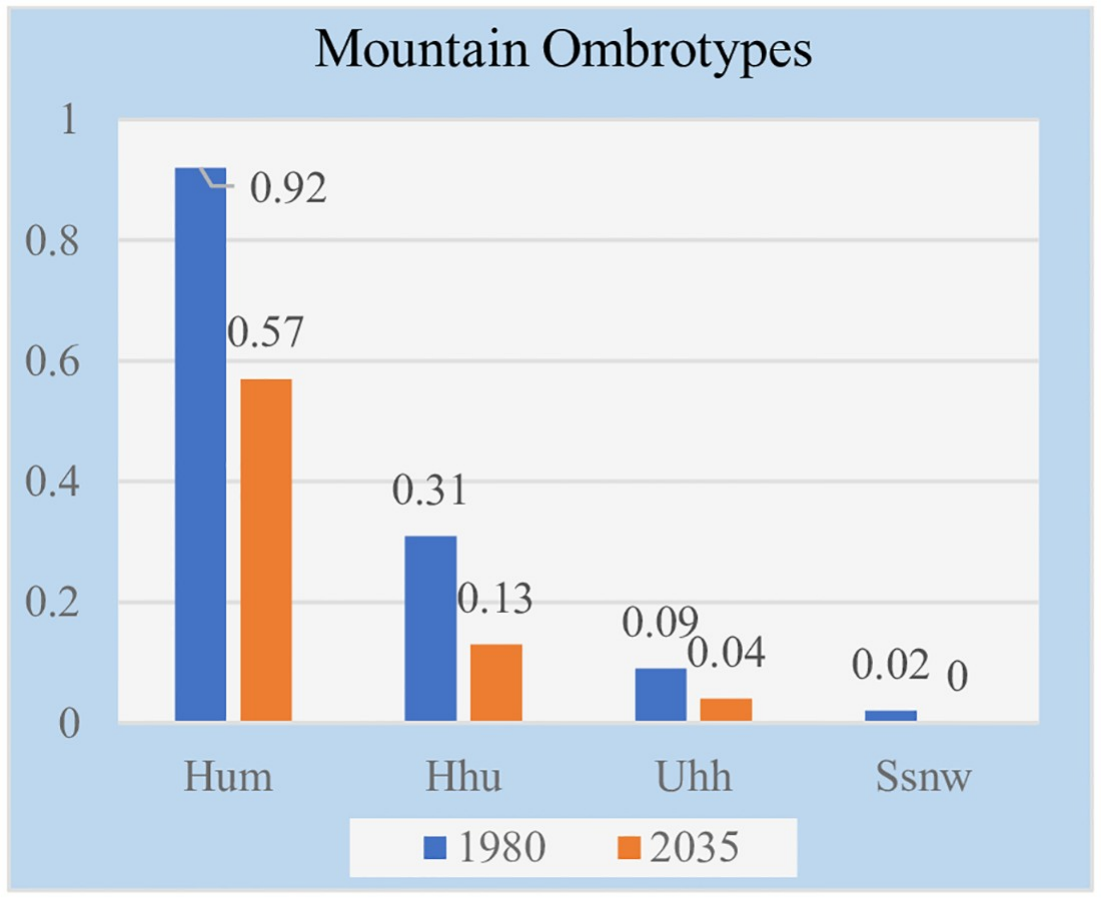
Mountain ombrotypes.

Impacts of projected climate and glacier change in the Northern Tien Shan, Kazakhstan, Central Asia [66] have been investigated using the regional climate model PRECIS driven by four different GCM-scenario combinations (HadGEM2.6, HadGEM8.5, A1B using HadCM3Q0 and ECHAM5). All climate scenarios showed statistically significant warming in the 21st Century. Neither projects show statistically significant change in annual precipitation. These results coincide with our bioclimatic assessment of Kazakhstan mountains. The TEXE, BOCO and BOXE bioclimates are found almost only in the mountainous zones, as well as the cold thermotypes Ome, Ote, Cte, Obo and Cbo, and the humid ombrotypes Hum, Hhu, Uhh, and Snw. All of these bioclimatic units occupy very small areas governed by height. In Tables 12 and 13 and in Figs 15 and 16, we collected the percentages of these mountain thermotypes and ombrotypes and graphically represented their values. We have found that bioclimate change also affects mountains: from the beginning to the middle of the 21st century, those mountain bioclimates and all mountain thermo- and ombrotypes decrease in area or even disappear, which yields an assessment of increases in thermicity and aridity, even in the mountains, and despite the increase in height.

**Table 12:** Mountain thermotypes.

Thermotypes	1980–2004		2035–2059		Losses	
	Grid cells	%	Grid cells	%	Grid cells	%
**Ote**	48	1.05	39	0.85	-9	-0.2
**Cte**	22	0.48	9	0.2	-13	-0.28
**Obo**	7	0.15	0	0	-7	-0.15
**Cbo**	7	0.15	0	0	-7	-0.15
**Gel**	1	0.02	0	0	-1	-0.02

**Table 13:** Mountain ombrotypes.

Ombrotypes	1980–2004		2035–2059		Losses	
	Grid cells	%	Grid cells	%	Grid cells	%
**Hum**	42	0.92	26	0.57	-16	-0.35
**Hhu**	14	0.31	6	0.13	-8	-0.18
**Uhh**	4	0.09	2	.,04	-2	-0.04
Ssnw	1	0.02	0	0	-1	-0.02

### Climatology / bioclimatology

Climate change is said to be global because the expected climate change affects, to a greater or lesser extent, the entire earth. But when the climate values predicted in global climate change are submitted to the WBCS bioclimatic analysis, it is surprisingly found that certain areas of the earth remain bioclimatically stable for one or more of the bioclimatic units: macrobioclimates, bioclimates, thermotypes, ombrotypes, isobioclimates. It is very different to climatically say that a general warming of the country is expected during the 21st century [69–71] than to affirm: 90.3% of the country maintains its macrobioclimate stable; 73.1% of the country maintains stable its bioclimate; 50.6% of the territory maintains its thermotype stable; 71.3% maintains its ombrotype stable; and 35.8% of the country maintains its isobioclimate stable. That is, despite the expected general climate change, there are many territories with bioclimatic stability. To assess bioclimatic stability in our study, we have analyzed the data collected in S4 Table for each bioclimatic indicator, for each study period, and for each of the 4569 points. (See Table 14). The predicted global climate change will not affect the geographical distribution of plant species, or their communities, in more than 1/3 of the Kazakh territory -Isobioclimates stability-.

**Table 14:** Bioclimatic stability.

Bioclimatic units between 1908–2059	BIOCLIMATIC STABILITY in %	BIOCLIMATIC CHANGE in %
Macrobioclimates	90.3	9.7
Bioclimates	73.1	26.9
Thermotypes	50.6	49.4
Ombrotypes	71.3	28.7
11 Major Isobioclimates	35.8	64.2

Very surprisingly, the bioclimatic analysis of the projected climate change has also allowed us to verify that the astonishing bioclimatic stability found is accompanied by a loss of bioclimatic diversity. That is, as we move into the 21st century, the number of elements in all the bioclimatic units decreases. In Table 15, we have compiled our results for all the bioclimatic units (see Tables 4, 5, 7, 8 and 9), together with the % of loss for each of the bioclimatic units. In Kazakhstan, according to PRECIS, there will be a generalized loss of bioclimatic diversity, that is to say, a generalized bioclimatic homogenization.

**Table 15:** Loss of bioclimatic diversity / bioclimatic homogenization.

Bioclimatic units	1st^st^-2nd Periods: there were … elements	3rd Period: there are2026 elements	N° of elements that disappeared	% Loss
Macrobioclimates	3	2	1	33.3
Bioclimates	**10**	5	**5**	**50.0**
Thermotypes	**11**	7	**4**	**36.3**
Ombrotypes	10	9	1	10.0
Isobioclimates	**43**	27	**16**	**37.2**

### Climate change, natural vegetation and agriculture

In a study on potential areas of deciduous forests in Castile and Leon (Spain) according to future climate change [72], bioclimate maps are also carried out on climate forecasts deduced from climatic trends observed in the last third of the 20th century, for three periods of the 21st century. And the foreseeable changes in the areas occupied by various deciduous forests in the region are analyzed. "The paper emphasizes the relevance of using bioclimatic models to anticipate possible changes in the natural vegetation of a territory." In other words, decision-makers will be able to use all our biolimatic maps to know the future extent and location of each bioclimatic situation, of each isobioclimate, and, with that, prepare, forecast everything needed for the new locations of the crops.

### Comparison with other climatic and zoning maps of Kazakhstan

**Fig 17:**
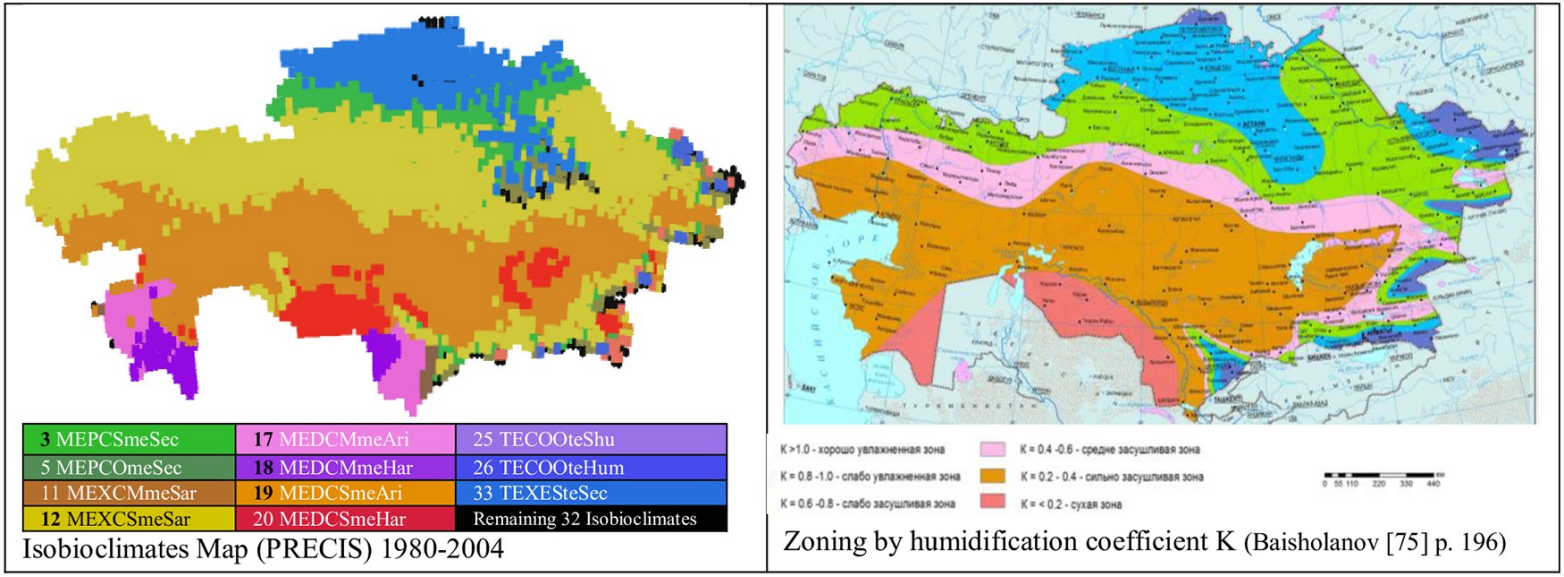
Comparison of isobioclimates map and Baisholanov humidification map. (Isobioclimates map, our own elaboration for this article; “zoning by humidification coefficient K” map, reprinted from [75] under a CC BY license, with permission from Baisholanov, original copyright [2017]).

Our PRECIS isobioclimates map corresponding to the period 1980–2004, shown in Fig 17, has been compared with several climatic maps and zoning maps of Kazakhstan. There are similarities in the distribution of the bioclimate polygons built according to the Rivas-Martinez method and the zones and isolines of some maps from the Atlas of Kazakhstan [73], for example, the maps "Natural-agricultural zoning", "Soil-geographical zoning", "The moisture availability", "Vegetation cover" and "Ecosystem map". However, our “Isobioclimates PRECIS 1980–2004” map is still more similar to the maps "Agroclimatic zoning" and "The moisture availability of the vegetation period" [74] and, overall, with the "Zoning by humidification coefficient K" map [75] (Fig 17).

Both maps, the Baisholanov map "Zoning by humidification coefficient K" [75] and our “PRECIS 1980–2004 Isobioclimates map”, show striking similarities with respect to the general distribution of climatic and bioclimatic units. However, the two maps also present some differences: thus, Baisholanov distinguishes 6 zones by their level of humidification, while in our “PRECIS 1980–2004 Isobioclimates Map”, we find 11 isobioclimates. This is because we take into account the thermal level and the annual rhythm of rainfall, in addition to the humidity level.

However, we must not forget that our “PRECIS 1980–2004 Isobioclimates map” is based on data from the PRECIS prediction, while the maps from the Atlas of Kazakhstan, such as those from Baisholanov’s publications, reflect real data from agricultural observations or climate data collected at meteorological stations. It would be very convenient to produce a "Bioclimatic Atlas of Kazakhstan 1980–2004" based on real data from meteorological stations, with maps of all possible bioclimatic units, and compare these maps with the current distribution maps of the agricultural species. In this way, we would truly understand the bioclimatic requirements of Kazakhstan agricultural species.

### Comparison between bioclimates maps from historical data and from PRECIS projection data 1980–2004

**Fig 18:**
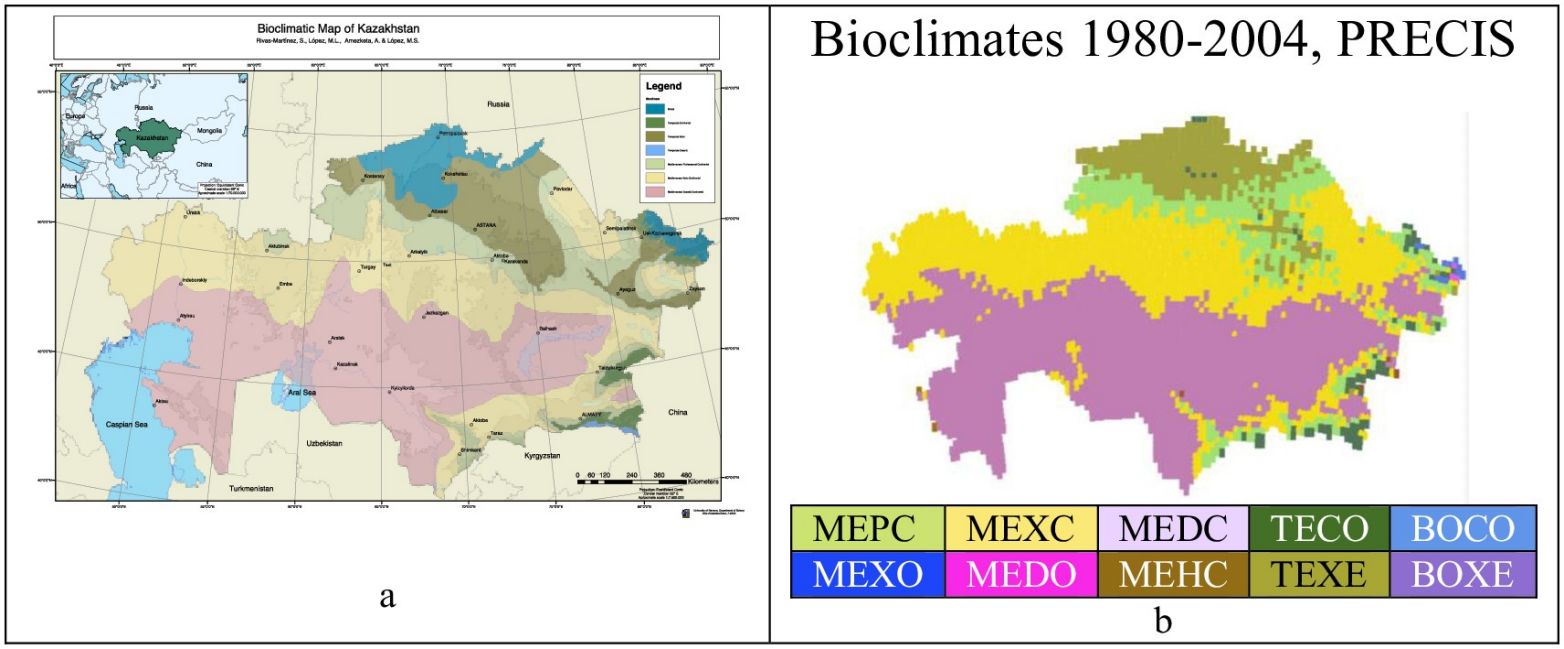
Historical and PRECIS projection bioclimatic maps. (a, “reprinted from [64], under a CC BY license, with permission from ML Lopez Fernandez, original copyright 2018”; b, our own elaboration for this article).

Taking into account all the available meteorological data for Kazakhstan from the end of the 19th century until 1968 [54], a bioclimatic map of Kazakhstan was published [42, 64]. Although our work now uses prediction data from PRECIS, we find it interesting to compare this “historic bioclimates map” with the one corresponding to the years 1980–2004 of the PRECIS projection (see Table 16 and Fig 18). In both maps, the same bioclimates appear, with the exception of MEXO and MEHC, which are missing from the historical map, and of TEOC, which is missing in the PRECIS data. Additionally, in both maps, the MEDC bioclimate forms an extensive latitudinal band in the central and SSE parts of the country and is surrounded to the N and to the SSE by another band of MEXC; wrapping MEXC, also to the N and to the SSE, there is another MEPC band (discontinuous in the SSE). In both maps, the bioclimate TEXE appears only in the N and NE, and TECO appears in the mountains of the SSE.

**Table 16:** Differences between the historical data bioclimates map and our PRECIS 1980–2004 bioclimates map.

Bioclimates	Historical data map	1980–2004 PRECIS map
MEPC	+	+
MEXO	-	+
MEXC	+	+
MEDC	+	+
MEHC	-	+
TEOC	+	-
TECO	+	+
TEXE	+	+
BOCO	Boreal +	+
BOXE		+

Regarding the differences, the most striking change is the enlargement of the MEDC area to the SE and E to contact the Chinese border in the depression of Lake Alakol, occupying areas that were previously MEXC and MEPC, but a large part of the depression of Lake Zaysan is also changed to MEDC. The MEXC loses ground in the SE but gains in the NE, where it occupies areas that were previously MEPC and TEXE. The historical MEPC moves towards the N part of the country, where it occupies previous positions of TEXE and even boreal; in addition, at the NE end, it loses much area in favor of MEXC and even of MEDC. In the PRECIS projection, we note the disappearance of TEOC; TECO maintains its positions and expands with some representation along the S edge of the country and occupies some positions in the flanks of the Lake Zaysan depression and other positions abandoned by the boreal in the N and NE parts of the country; at the same time, historical TEXE yields a significant part of its area to MEPC, a loss that is compensated in part because it gains almost the entire area of the historical BOREAL that occupies the extreme N central part; in addition, it disappears completely on the edges of the Lake Zaysan depression. Finally, BOREAL, BOCO and BOXE disappear completely on the N edge of the country, where they are replaced by TEXE and some TECO, and almost disappear from the NE end near Oskemen, where they are replaced by TECO, MEPC and MEXC. This shift of the boreal border has been confirmed in other studies. For example, in [76] it has been stated that increased temperature leads to a decrease in effective moisture, which in turn causes a decline in growth and a lack of regeneration of boreal forests.

In summary, MEDC expands towards the four cardinal points; MEXC expands to the N and NE; TEXE almost disappears in the NE and moves towards the N, where it occupies the place of BOREAL; and finally, BOREAL disappears in the N and remains very diminished or vestigial in the NE. The same trends operate that we see later in the course of the 21^st^ century.

## Conclusions

Both we and the various authors mentioned in the DISCUSSION, using different climate models, found similar trends for climate change in Kazakhstan in the 21st century: Widespread warming, more intense in summer, and slight decrease in rainfall. Bioclimatically, these climatic projections produce the following consequences:

- Increase in thermicity in 36.2% of the territory,
- Increase in continentality, in 7.3% of the territory,
- Increase in annual aridity, in 10.3% of the territory,
- Increase in Mediterraneity, in 9.5% of the territory,
- In the mountains, disappearance of all three mountain bioclimates, and also decrease of the area, or even disappearance, of all mountain thermotypes and ombrotypes.
- Although climate change affects the whole of Kazakhstan, 35.8% of the country maintains total isobioclimatic stability, none of the bioclimatic indicators change.
- As we move into the 21st century, there is a loss of bioclimatic diversity: the number of macrobioclimates decreases by 33.3%; that of bioclimates, 50.0%; that of thermotypes, 36.3%; that of ombrotypes, 10.0% and that of isobioclimates, 37.2%. That is to say, there will be a generalized bioclimatic homogenization.
- Bioclimatically, Kazakhstan is primarily a strongly continental country, mostly mediterranean and steppe, with mainly mediterranean-continental bioclimates MEDC, MEXC and MEPC, cold thermotypes (supramediterranean Sme and mesomediterranean Mme), and arid and semiarid ombrotypes (Ari and Sar). The rest of the bioclimatic unit values have a merely vestigial territorial representation, since they occupy very small areas.
- We strongly recommend developing a "Bioclimatic Atlas of Kazakhstan 1980–2014" with real meteorological data to be compared with the current distribution maps of the cultivated species in order to define the "Bioclimatic Requirements" of the Agricultural Species in Kazakhstan.

## Supporting information

S1 Fig Bioclimatic synopsis of the earth. (TIFF)

S2 Fig Seasonal mean winter and summer temperature validation. 1980–2004.
(TIF)

S3 Fig Seasonal mean precipitation validation. 1980–2004.
(TIF)

S1 Table Data 1980–2004. (ODS)

S2 Table Data 2010–2034. (ODS)

S3 Table Data 2035–2059. (ODS)

S4 Table Bioclimatic identification. (ODS)
