# Supplementary figures and images for: Assessment of bioclimatic change in Kazakhstan, end 20th—middle 21st centuries, according to the PRECIS prediction
Source: PLoS One. 2020 Oct 2;15(10):e0239514. doi: 10.1371/journal.pone.0239514 (PMC7531827; doi:10.1371/journal.pone.0239514)

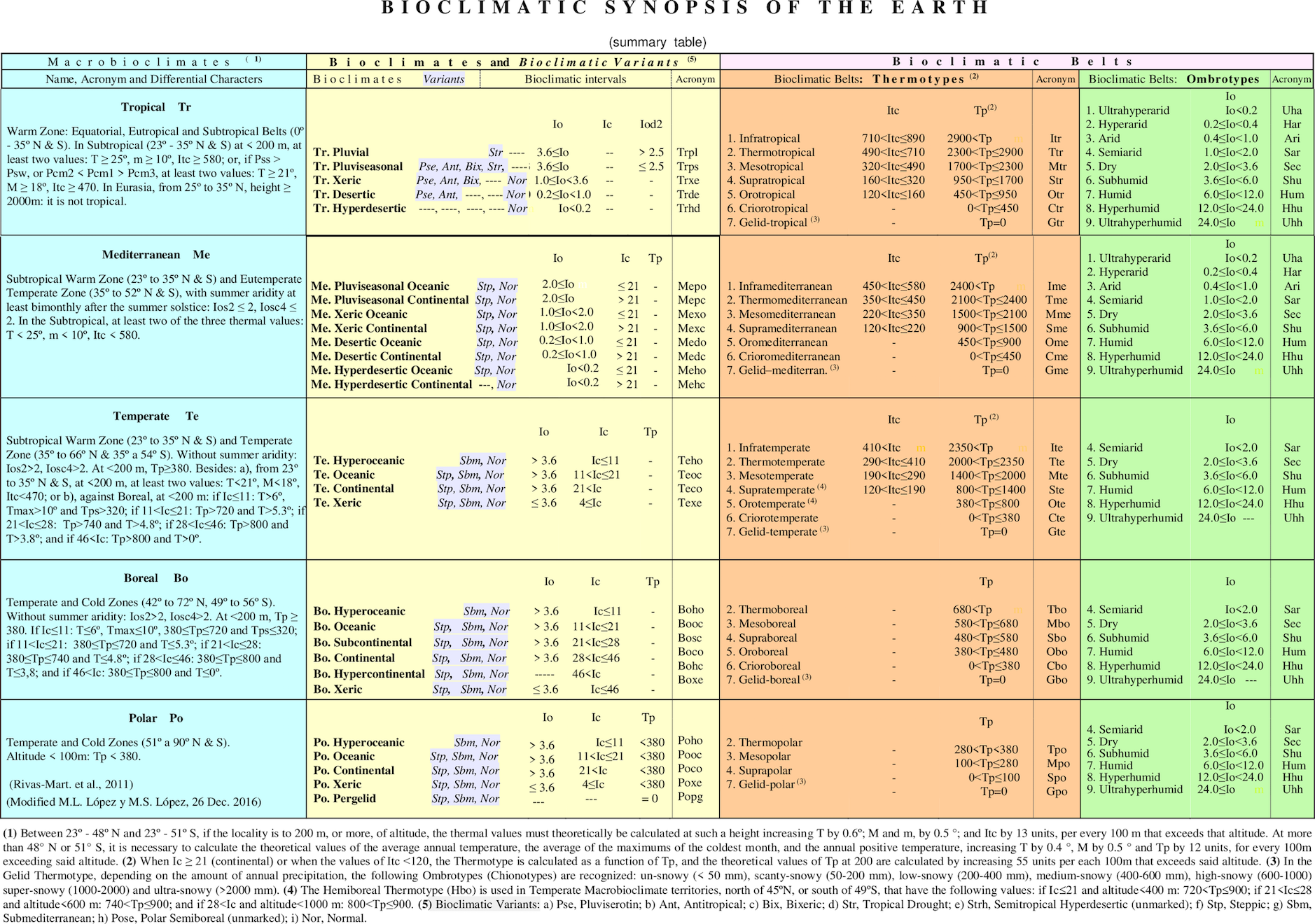

Supplement: S1 Fig — (TIFF) [file pone.0239514.s001.tiff]

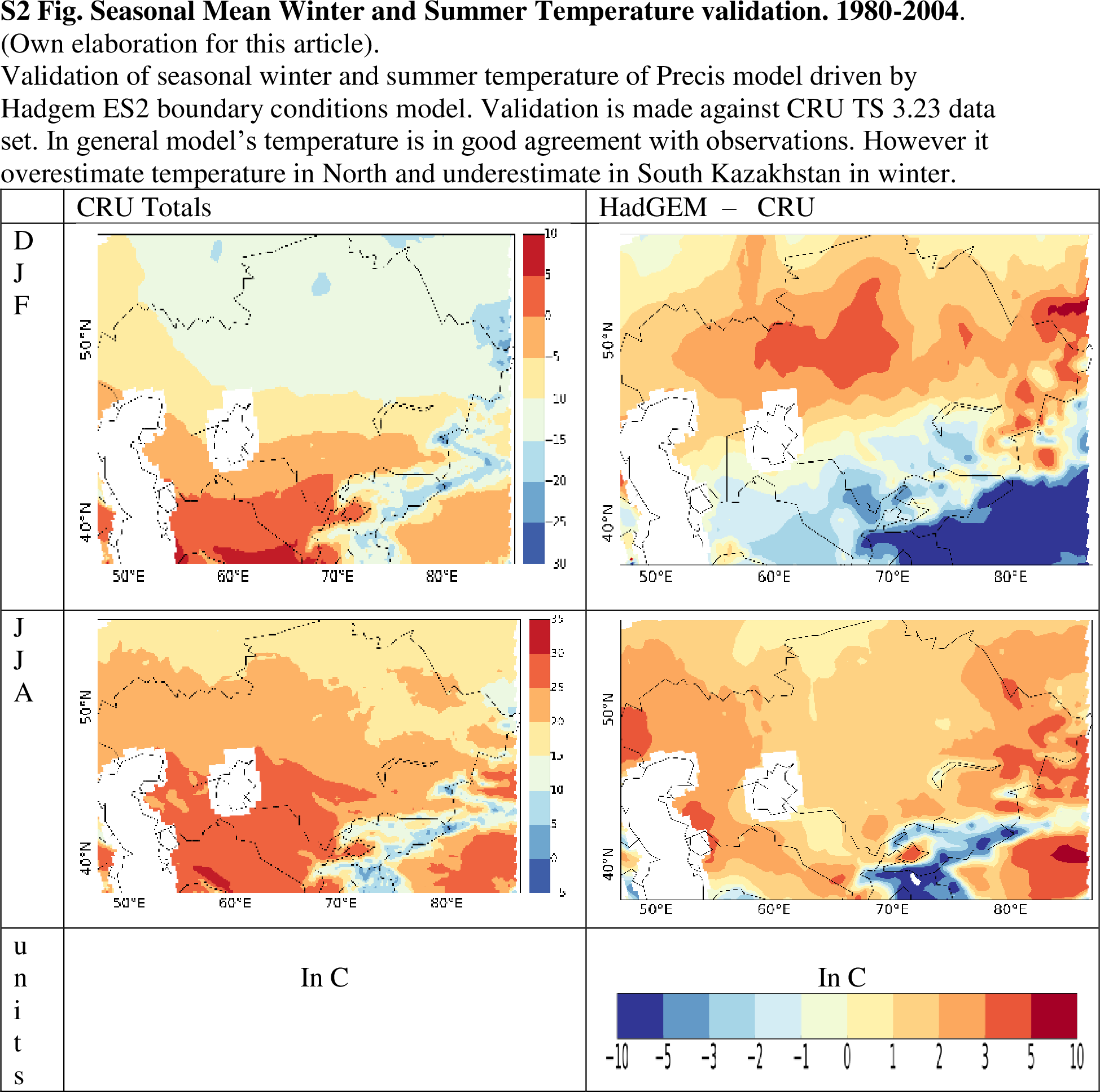

Supplement: S2 Fig — 1980–2004. (TIF) [file pone.0239514.s002.tif]

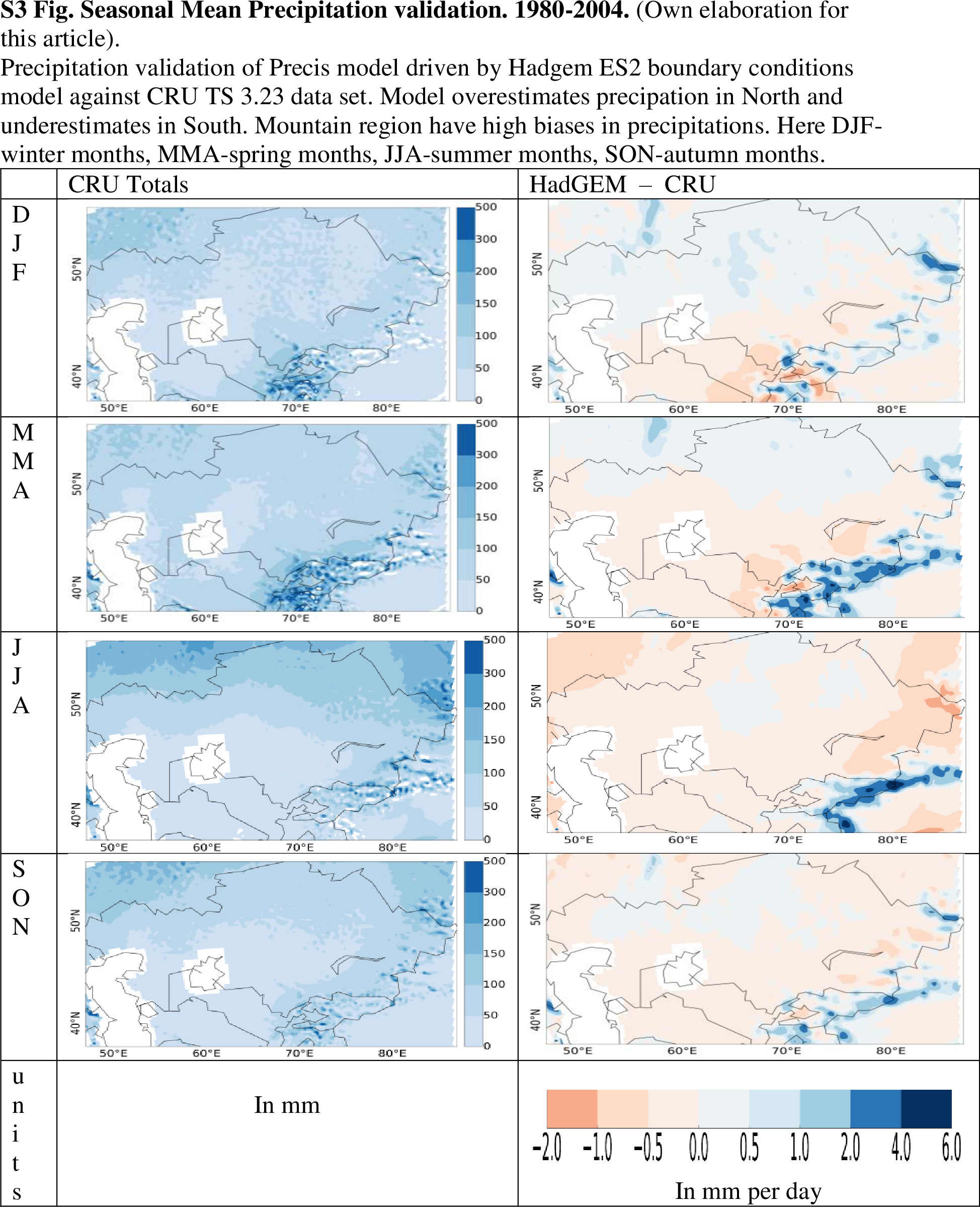

Supplement: S3 Fig — 1980–2004. (TIF) [file pone.0239514.s003.tif]
